# Global Interactome Mapping Reveals Pro-tumorigenic Interactions of NF-κB in Breast Cancer

**DOI:** 10.1016/j.mcpro.2024.100744

**Published:** 2024-02-28

**Authors:** Petr Lapcik, R. Greg Stacey, David Potesil, Petr Kulhanek, Leonard J. Foster, Pavel Bouchal

**Affiliations:** 1 Department of Biochemistry, Faculty of Science, Masaryk University, Brno, Czech Republic; 2 Michael Smith Laboratories, University of British Columbia, Vancouver, Canada; 3 Proteomics Core Facility, Central European Institute of Technology, Masaryk University, Brno, Czech Republic; 4 National Centre for Biomolecular Research, Faculty of Science, Masaryk University, Brno, Czech Republic; 5 Department of Biochemistry and Molecular Biology, University of British Columbia, Vancouver, Canada

**Keywords:** NF-κB, RELA, proteomics, protein complexes, interaction, breast cancer, protein correlation profiling, AlphaPullDown

## Abstract

NF-κB pathway is involved in inflammation; however, recent data shows its role also in cancer development and progression, including metastasis. To understand the role of NF-κB interactome dynamics in cancer, we study the complexity of breast cancer interactome in luminal A breast cancer model and its rearrangement associated with NF-κB modulation. Liquid chromatography-mass spectrometry measurement of 160 size-exclusion chromatography fractions identifies 5460 protein groups. Seven thousand five hundred sixty eight interactions among these proteins have been reconstructed by PrInCE algorithm, of which 2564 have been validated in independent datasets. NF-κB modulation leads to rearrangement of protein complexes involved in NF-κB signaling and immune response, cell cycle regulation, and DNA replication. Central NF-κB transcription regulator RELA co-elutes with interactors of NF-κB activator PRMT5, and these complexes are confirmed by AlphaPulldown prediction. A complementary immunoprecipitation experiment recapitulates RELA interactions with other NF-κB factors, associating NF-κB inhibition with lower binding of NF-κB activators to RELA. This study describes a network of pro-tumorigenic protein interactions and their rearrangement upon NF-κB inhibition with potential therapeutic implications in tumors with high NF-κB activity.

## Highlights

• SEC-PCP-SILAC mapping of MCF-7 protein complexes with inhibited/uninhibited NF-κB.
• Comprehensive network of 7568 interactions among 1520 proteins.
• Described impact of NF-κB inhibition on pro-tumorigenic complexes.
• Novel interaction partners of RELA and its activator PRMT5.
• Protein complex validation using AlphaPulldown.

## Graphical Abstract

**Figure:**
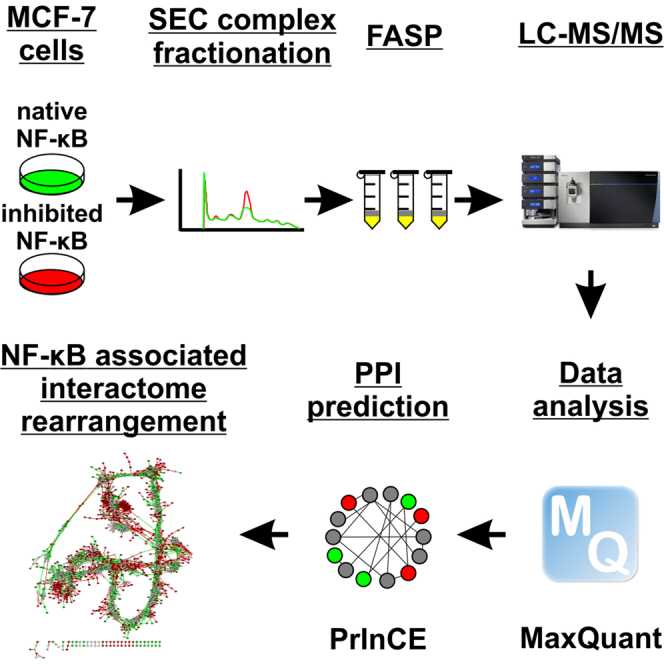

## In Brief

Lapcik *et al*. apply SEC-PCP-SILAC mapping of protein complexes to describe influence of NF-κB activity on interactome of breast cancer cells. The resulting network of 7568 interactions that includes 1520 proteins detects remodeling of complexes involved in immune response as well as pro-tumorigenic mechanisms including cell cycle regulation. This interactome dataset is ready to serve as a data source to target the interactions that may have a therapeutic implications in breast cancer.

Family of NF-κB transcription factors is present in all cell types and is known as a key regulator of inflammation and innate immune response (1, 2, 3, 4). NF-κB pathway involves five key proteins, RELA (p65), RELB, c-Rel, NFKB1 (p50), and NFKB2 (p52), that occur in a form of homodimers or heterodimers (5). In nonstimulated cells, NF-κB protein complexes interact with proteins of IκB family that prevent from transition of NF-κB dimers from cytosol into the nucleus, thus inhibiting transcriptional regulation of NF-κB target genes (6, 7). The NF-κB pathway is triggered by a variety of growth factors, pro-inflammatory cytokines, and signaling pathways (2, 8, 9). Stimulation of NF-κB leads to activation of IκB kinase (4) and subsequent degradation of IκB protein. NF-κB subunit released from inhibitory complex is transported into the nucleus, binds target sequences, and activates transcription of relevant genes (2, 3, 4). In cells of the immune system, activation of NF-κB pathway results in the production of inflammatory cytokines, including TNFα, IL-1, IL-6, and IL-8, chemokines, and cell survival and proliferation factors (10). NF-κB also supports survival of cells inflicted with a stress from inflammation by enhancing expression of anti-apoptotic genes (2).

Nevertheless, former studies linked aberrant activity of NF-κB with cancer, including enhanced expression of NF-κB factors in breast cancer cells as well as their participation in breast tumorigenesis (9, 11, 12, 13, 14, 15). Our previous studies (16, 17) identified association between increased transcript and protein NF-κB levels and lymph node metastasis of luminal A breast tumors. Inhibition of NF-κB thus hypothetically represents a potential anti-metastatic therapeutic strategy for this group of tumors.

To deeply understand the cellular pathways associated with NF-κB in breast tumors, the global mapping of protein–protein interactions dependent on NF-κB status could introduce information elusive for other methods commonly used in cancer research. However, generation of complex differential protein–protein interaction networks is a challenging task (18). Up to date, no studies aimed to unravel the impact of NF-κB activity on protein interactome on the proteome scale so far. The state-of-art method of choice abbreviated as SEC-PCP-SILAC is based on stable isotope labeling by amino acids in cell culture (SILAC), separation of native protein complexes by size-exclusion chromatography (SEC), and protein correlation profiling (PCP) (19). SEC separates protein complexes by their size, and protein–protein interaction probability is computed according to co-elution of proteins in SEC fractions. Triplex SILAC labeling is used to compare interaction networks under different conditions. This untargeted technique does not require any protein tagging, fusion, or overexpression and allows to measure the interactome rearrangements in response to cellular stimuli (19). Machine learning computational methods aim at identification of protein interactions based on protein SEC chromatograms (20). SEC-PCP-SILAC thus represents a powerful approach for mapping thousands of protein interactions in one experiment and reconstructing complex interaction networks in the human protein interactome (21). In this study, we hypothesize that SEC-PCP-SILAC is able to map the pro-tumorigenic NF-κB protein–protein interactions and their response to NF-κB modulation in MCF-7 cell line derived from luminal A breast tumor. The resulting interactions were validated in independent datasets and using AlphaPulldown *in silico* analysis, providing a substantial extension of knowledge on the reconstruction of MCF-7 protein interactome related to suppression of NF-κB pathway. The interactome network is ready to serve as a data source to target the interactions that may have therapeutic implications.

## Experimental Procedures

### Experimental Design and Statistical Rationale

For the SEC-PCP-SILAC experiment, one biological replicate labeled with SILAC and one label-free replicate were generated. For each biological replicate, NF-κB inhibition using transfection of MCF-7 breast cancer cell line with plasmid encoding NFKBIA was performed in parallel with transfection by empty plasmid as a control. Each replicate was separated using SEC in 80 fractions that were measured using data-dependent acquisition (DDA) LC-MS/MS resulting in 160 runs in total. We used the MaxQuant (22) software downloaded from https://www.maxquant.org/ to create elution profiles for all individual proteins in each of the SEC datasets and applied a Prediction of Interactomes from Co-Elution (PrInCE) (20) algorithm to predict protein–protein interactions. To confirm co-elutions of discussed proteins, a third SEC biological replicate consisting of additional 34 SEC fractions of cells with no transfection was prepared. Moreover, immunoprecipitation of NF-κB factor RELA in six biological replicates and analysis of total proteome changes after NF-κB inhibition in four biological replicates were carried out, for both data were acquired using data-independent acquisition (DIA) LC-MS/MS.

### Cell Culture

MCF-7 breast cancer cells were obtained from Sigma-Aldrich and used for the experiments. For preparation of the SILAC labeled replicate for the SEC-PCP-SILAC experiment, MCF-7 cell line was grown in SILAC Advanced Dulbecco’s modified Eagle’s medium (DMEM)/F12 Flex Media (Lys/Arg ^−/−^, Thermo Fisher Scientific) supplemented with self-dialyzed 10% fetal bovine serum (FBS, Thermo Fisher Scientific, 3.5 kDa MWCO), 1% Penicillin/Streptavidin (Sigma-Aldrich), 4 mM L-glutamine (Sigma-Aldrich), 0.5 μg/ml hydrocortisone (Sigma-Aldrich), 10 μg/ml insulin (Sigma-Aldrich), 20 ng/ml epidermal growth factor (Thermo Fisher Scientific), 4 mg/l D-glucose (Sigma-Aldrich), 50 mg/l proline (Sigma-Aldrich), and following combinations of lysine and arginine: for the light (L) SILAC population, L-lysine (25 mg/l, Thermo Fisher Scientific) and L-arginine (25 mg/l, Thermo Fisher Scientific); for the medium (M) SILAC population, D_4_-L-lysine (25 mg/l, Thermo Fisher Scientific) and ^13^C_6_-L-arginine (25 mg/l, Thermo Fisher Scientific); for the heavy (H) SILAC population, ^13^C_6_-^15^N_2_-L-lysine (25 mg/l, Thermo Fisher Scientific) and ^13^C_6_-^15^N_4_-L-arginine (25 mg/l, Thermo Fisher Scientific). Cells were grown under 5% CO_2_ and 37 °C. Cells were passaged after reaching 80% confluence. Growth medium was removed from the plates; cells were washed with 0.5% EDTA in PBS (0.137 M NaCl; 2.68 mM KCl; 1.47 mM KH_2_PO_4_; 6.45 mM Na_2_HPO_4_) and treated with 0.125% trypsin solution (Sigma-Aldrich) at 37 °C for 3 to 6 min. Passaged cells were washed with a complete medium and transferred on new plates. SILAC labeling was performed during 10 cell passages to ensure sufficient incorporation of isotope-labeled amino acids. One day before the transfection, cells for each SILAC channel were passaged on 4 × 15 cm plates to reach 50% confluency at the time transfection. For preparation of the label-free part (SEC-label-free) for the SEC-PCP-SILAC experiment, MCF-7 cells were grown in DMEM media (Sigma-Aldrich) supplemented with 10% FBS under 5% CO_2_ and 37 °C. One day prior to the transfection, cells were seeded on 8 × 15 cm plates to reach 50% confluency at the time of transfection—four plates for the cells with inhibited NF-κB pathway and four plates for the mock cell line.

For immunoprecipitation experiment, MCF-7 cells were grown in DMEM media supplemented with 10% FBS under 5% CO_2_ and 37 °C. One day prior to the transfection, cells were seeded on 24 × 15 cm plates to reach 50% confluency at the time of transfection—12 plates for the cells with inhibited NF-κB pathway and 12 plates for the mock cell line, one plate per biological replicate.

### Plasmids and Cell Transfection

Plasmid encoding Flag-tagged IκBα (pCMV4-FLAG-ΔN IκBα), which contains the human IκBα gene NFKBIA with deletion of N-terminal 36 amino acids to prevent its phosphorylation and thus its degradation, was described previously (23). Cell transfection for each replicate for SEC-PCP-SILAC experiment was performed as follows: 48 μg of pCMV4-FLAG-ΔN IκBα or empty pCMV4 plasmid and 144 μg of PEI was dissolved in 8 ml of blank SILAC Advanced DMEM/F12 Flex Media or DMEM media, vortexed, and incubated for 15 min at RT. Two milliliters of plasmid-PEI mixture per 15 cm (i.d.) plate was added (pCMV4-FLAG-ΔN IκBα plasmid to the H population and label-free cells, empty pCMV4 plasmid to the M population and label-free cells, and empty medium to the L population). The cells were incubated for 36 h. For the cells for immunoprecipitation experiment, the cells were transfected with pCMV4-FLAG-ΔN IκBα or empty pCMV4 plasmid as described above, and the cells were incubated for 36 h.

### Cell Harvesting and Preparation of Native Protein Complexes for SEC-PCP-SILAC Analysis

Preparation of SEC-PCP SILAC-labeled and label-free replicates and subsequent SEC fractionation were carried out according to (19) with modifications. Medium was removed from the plates and cells were washed three times with ice cold 1× PBS. Dishes with cells were then kept on ice. Cells were scraped in the ice-cold SEC mobile phase (50 mM Tris, 50 mM KCl, 50 mM Na-acetate, pH 7.2) supplemented with cOmplete, EDTA-free Protease Inhibitor Cocktail (Roche). Aliquots representing 10% of the volumes of the label-free cell suspensions were removed for the total proteome analysis. Cells of the same SILAC population or label-free cell populations transfected with the same plasmid were pooled together. Cell lysis was performed using Dounce tissue grinder for 4 min. The homogenates were then centrifuged at 100,000*g* and 4 °C for 15 min, and supernatants were concentrated using the 100 kDa molecular weight cutoff spin columns (Sartorius Vivaspin). Equal volumes of H and M lysates were mixed and further subjected to SEC fractionation. Protein complexes of L lysate and label-free populations were fractionated separately.

### SEC Fractionation of Protein Complexes

Separation of protein complexes was performed on Agilent 1100 liquid chromatograph (Agilent Technologies) using 50 × ProSEC 300S 50 × 7.5 mm pre-column online connected to two serially connected ProSEC 300S 300 × 7.5 mm columns (all Agilent Technologies) equilibrated with the SEC mobile phase. Mixture of standards (Protein Standard Mix 15–600 kDa, Sigma-Aldrich) with added bovine serum albumin (66 kDa, Bio-Rad) was used for calibration of SEC columns. Separation was carried out at a flow rate of 0.5 ml/min at 4 °C. Eighty fractions from mixed H/M lysates were collected from 20 to 60 min (2 fractions/min). L lysate was separated on 20 fractions that were pooled and evenly distributed to the H/M fractions as internal standard. Each of the label-free lysates was fractionated to 30 fractions from the 20 to 40 min (1.5 fractions/min) and 10 fractions from the 40 to 60 min (0.5 fractions/min), 40 fractions in total. Protein concentrations in all fractions were determined using Qubit Protein Assay Kit (Invitrogen). Samples were subjected to trypsin digestion using modified filter-aided sample preparation method (24) and desalted as previously described (25). Briefly, proteins from the whole combined fractions were transferred to the Microcon filter device, cut-off 30 kDa (Millipore), reduced by tris(2-carboxyethyl)phosphine) (Sigma-Aldrich) in 8 M urea in 0.1 M Tris/HCl, pH 8.5, alkylated using iodoacetamide (Sigma-Aldrich), digested by trypsin (Promega) with the ratio trypsin:protein 1:30 at 37 °C overnight, and resulting peptides were desalted on MicroSpin columns C18 (Nest Group).

### LC-MS/MS Analyses for SEC-PCP-SILAC in DDA Mode

Lyophilized peptides were extracted into LC-MS vials by 2.5% formic acid (FA) in 50% acetonitrile (ACN) and 100% ACN with addition of PEG (20,000; final concentration 0.001%) (26) and concentrated in a SpeedVac concentrator (Thermo Fisher Scientific). Peptide concentration in the peptide concentrates was ascertained using quality control LC-MS run using RSLCnano system (Thermo Fisher Scientific) connected to HCT Ultra ion trap mass spectrometer (Bruker).

LC-MS/MS analyses of all peptide mixtures of the SILAC-labeled replicate were done using RSLCnano system connected to Orbitrap Fusion Lumos mass spectrometer (Thermo Fisher Scientific). Mixture of iRT peptides (Biognosys) were spiked into all samples prior the measurement. Prior to LC separation, tryptic digests (approx. 2 μg of peptides) were online concentrated and desalted using trapping column (100 μm × 3 cm, 3.5 μm X-Bridge BEH 130 C18 sorbent, Waters; temperature of 40 °C). After washing of trapping column with 0.1% FA, the peptides were eluted (flow rate - 300 nl/min) from the trapping column onto an analytical column (Acclaim Pepmap100 C18, 3 μm particles, 75 μm × 500 mm; at temperature of 40 °C, Thermo Fisher Scientific) by 109 min linear gradient program (5–37% of mobile phase B; mobile phase A: 0.1% FA in water; mobile phase B: 0.1% FA in 80% ACN). Equilibration of the trapping column and the analytical column was done prior to sample injection to sample loop. The analytical column outlet was directly connected to the Digital PicoView 550 (New Objective) ion source with sheath gas option and SilicaTip emitter (New Objective; FS360-20-15-N-20-C12) utilization. Active Background Ion Reduction Device (ABIRD), ESI Source Solutions was installed.

MS data were acquired in a DDA strategy with cycle time for 3 s and with survey scan (350–2000 m/z). The resolution of the survey scan was 60,000 (200 m/z) with a target value of 4 × 10^5^ ions and maximum injection time of 50 ms. HCD MS/MS (30% relative fragmentation energy, normal mass range) spectra were acquired with a target value of 5.0 × 10^4^ and resolution of 30,000 (200 m/z). The maximum injection time for MS/MS was 54 ms. Dynamic exclusion was enabled for 60 s after one MS/MS spectra acquisition. The isolation window for MS/MS fragmentation was set to 1.6 m/z.

LC-MS/MS analysis of peptide samples from the SEC-label-free dataset was performed using the RSLCnano system (Thermo Fisher Scientific) online connected to Impact II Ultra-High Resolution Qq-Time-Of-Flight (Bruker) mass spectrometer. Peptides were preconcentrated online on a 100 μm × 30 mm trapping column packed with 3.5-μm X-Bridge BEH 130 C18 (Waters) prior to LC separation. Equilibration of the trapping and analytical column was performed before the sample injection. Peptides were separated using an Acclaim Pepmap100 C18 column (3 μm particles, 75 μm × 500 mm, Thermo Fisher Scientific) using the following LC gradient (mobile phase A: 0.1% FA in water, mobile phase B: 0.1% FA in 80% ACN: 300 nl/min; 40 °C): Elution gradient started at 1% of mobile phase B, which increased to 56% over 120 min nonlinearly (40 min: 14%, 80 min: 30%, 120 min: 56%) followed by the system wash phase. Analytical column outlet was connected to the CaptiveSpray nanoBooster ion source (Bruker). NanoBooster was filled with acetonitrile. MS and MS/MS spectra were measured in DDA mode with 3 s long cycle. The mass range was set to 150 to 2200 m/z with precursors selection from 300 to 2000 m/z. Measurement frequency of MS and MS/MS scans were 2 Hz and 4 to 16 Hz (depending on the precursor intensity).

### LC-MS/MS Data Processing for SEC-PCP-SILAC Analysis

Protein identification and quantification was performed in MaxQuant 2.0.3.0 software (22). Database search was performed against human UniProt/SwissProt database (version 2021_03 downloaded on 2021-09-11, 20,371 sequences) using default settings for Orbitrap Lumos or Impact II mass spectrometer. Enzyme specificity was set to trypsin/P, two missed cleavages were allowed, fixed modifications were set to carbamidomethylation (C), and variable modifications were set to oxidation (M) and acetylation (protein N-terminus). Main search precursor mass tolerance was set to 4.5 ppm for SILAC and 0.006 Da for label-free dataset, respectively, and MS/MS mass tolerance was set to 20 ppm (FTMS) and 40 ppm (TOF). Match between runs and re-quantification were activated. FDR at peptide and protein levels were set to 0.01. For SEC-label-free replicate, label-free quantification was enabled. Potential contaminants, reverse hits, and peptides identified only based on posttranslational modifications were removed from the search results. Visualization of the MS/MS spectra was performed in the MS-Viewer tool (27) and the extracted ion current chromatograms were visualized in Skyline software version 22.2.0.351 (28). The SEC co-elution chromatograms were visualized in GraphPad software, version 9.3.1 (www.graphpad.com).

### Protein-Protein Interaction Network Reconstruction

Binary protein–protein interactions were computed from the SEC-PCP-SILAC profiles of SILAC-labeled and SEC-label-free replicates using the PrinCE algorithm (20) that reconstructs SEC chromatograms for individual proteins and calculates interaction score based on protein abundance in SEC fractions. The CORUM database (29) was used to discriminate true positive (TP) and false positive (FP) interactions. The precision was calculated for each protein–protein interaction in the network based on the ratio of TP to TP and true false among interactions of equal or higher probability. Interactions with precision at 50% or higher were included in our interaction dataset. The NF-κB–inhibited and control network comparisons in the terms of protein and interaction numbers, as well as protein co-elution visualization and heatmap construction of co-eluting subunits of known protein complexes from subset of CORUM database resolvable with SEC (30) were performed in the GraphPad Prism (version 9.0.1) software. Differential network comparing complete NF-κB–inhibited and NF-κB–uninhibited networks were visualized in Cytoscape software (version 3.8.2) downloaded from www.cytoscape.org (31) using DyNet application (version 1.0.0) (32). The detected protein–protein interactions were validated using CORUM, STRING v11.5 (33), GeneMania (34), HumanNet v3.0 (35), BioGrid (36), Reactome (37), IntAct (38), HIPPIE (39), IID (40), MINT (41), HINT (42), BioPlex 3.0 (43), and hu.MAP2.0 PPIs (44) databases, as well as the consensual human co-fractionation interaction dataset (45) and several other interactomics studies (21, 46, 47, 48, 49, 50, 51).

### Validation of the Biological Relevance of the NF-κB Interactome

Validation of the biological relevance of the protein–protein interaction network reconstructed by the PrInCE algorithm from the SEC-PCP-SILAC co-elution data was performed by assembling an aggregate network including unique interactions found either in NF-κB–inhibited or NF-κB–uninhibited interaction networks at 50% precision and comparing the aggregate networks to randomly rewired networks as described in (21). The Human Gene Ontology (GO) annotations, including biological process, molecular function, and cellular compartment, were processed and the total proportion of interacting protein pairs sharing at least one GO term in each ontological category was calculated for both rewired and observed networks, and the empirical *p* value for the observed enrichment was calculated. The same procedure was performed for tendency of interacting protein pairs to be associated with the same disease and the tendency of interacting protein pairs to contain domains known to physically interact in a high-resolution three-dimensional structure. Known interaction partners of RELA protein were searched in BioGRID, CORUM, HINT, HIPPIE, IID, InBioMap (52), MENTHA (53), MINT, and PINA (54) databases.

### Functional Analysis of NF-κB Interactome

Functional differences between interactomes with inhibited and uninhibited NF-κB pathway were evaluated according to (21). Briefly, for each GO term, annotated proteins were identified, for which number of interactions in NF-κB–inhibited or NF-κB–uninhibited interaction network was calculated as well as difference between the two networks. Networks were randomly rewired and difference in randomized networks was calculated. A z-score was calculated based on the null distribution of the randomized networks. The z-score was further converted to a probability and adjusted for multiple hypothesis testing. For comparison of the enriched GO terms to those based on the protein content of the NF-κB–inhibited and NF-κB–uninhibited networks, the odds of proteins in each network being annotated to the GO terms was calculated and tested for enrichment using the z score of the log-odds ratio. Significantly differentially enriched GO terms at 10% FDR were visualized as an enrichment map with Jaccard index cutoff set to 0.33.

### Immunoprecipitation

The NF-κB inhibited and mock cells subjected to immunoprecipitation analysis were washed three times with ice cold 1× PBS. Dishes with cells were then kept on ice. Cells were scraped in the ice-cold SEC mobile phase supplemented with cOmplete, EDTA-free Protease Inhibitor Cocktail (Roche). Cell lysis was performed using Dounce tissue grinder for 4 min. The homogenates were then centrifuged at 100,000*g* and 4 °C for 15 min, and supernatants were concentrated using the 100 kDa molecular weight cutoff spin columns (Sartorius Vivaspin). Protein concentration was determined by RC DC Protein Assay kit (Bio-Rad). Two hundred fifty micrograms of protein from NF-κB–inhibited or control cells was diluted to 250 μl in SEC mobile phase with protease inhibitors. Half of the replicates underwent pre-clearing with 25 μl of pre-washed Protein A/G Magnetic Beads (Thermo Fisher Scientific) and incubated for 1 h at 4 °C with rotation. Pre-cleared samples were transferred to new tubes. All samples were further incubated with rabbit anti-RELA (1:100; Cell Signaling Technology – cat. no. 8242) antibody or with control rabbit IgG (Dako, P0161) overnight at 4 °C with rotation. Twenty five microliters of pre-washed Protein A/G Magnetic Beads (Thermo Fisher Scientific) was added to the samples and incubated 1 h at 4 °C with rotation. Beads were washed three times with 500 μl of SEC mobile phase with protease inhibitors. Samples were then subjected to an on-bead in-solution trypsin digestion as follows: 0.5 μg of trypsin (Promega) in 50 μl of 25 mM ammonium bicarbonate was added to the beads and samples were incubated overnight at 37 °C. Supernatants were incubated with 5.6 μl of 100 mM tris(2-carboxyethyl)phosphine) for 30 min at 600 rpm and 37 °C. 2.94 μl of 300 mM iodoacetamide was added to the samples and the samples were alkylated for 1 min at 25 °C and 600 rpm and for 20 min in the dark without shaking. The samples were acidified with 0.59 μl of FA. Peptide samples were desalted on MicroSpin columns C18 (Nest Group) and dried under vacuum. The immunoprecipitation experiment was carried out in six biological replicates per condition.

### Sample Preparation for Total Proteome Analysis

For the total proteome analysis, cell suspension aliquots of NF-κB–inhibited or mock cell lines removed from each plate of the SEC-label-free replicate separately during the cell harvest representing 10% of the sample volume were resuspended in 8 M urea in 0.1 M Tris/HCl, pH 8.5. Samples were sonicated, incubated on ice for 1 h, and centrifuged at 14,000*g* and 4 °C for 20 min. Supernatants were transferred to new tubes. Protein concentration was determined by RC DC Protein Assay kit (Bio-Rad). Hundred microgrmas of protein from each sample was subjected to filter-aided sample preparation trypsin digestion and peptide desalting as described above.

### LC-MS/MS Measurements of Samples for Immunoprecipitation and Total Proteome Analysis in DIA Mode

LC-MS/MS analyses of samples from immunoprecipitation and total proteome experiment were done using RSLCnano system connected to Orbitrap Fusion Lumos mass spectrometer (Thermo Fisher Scientific). Mixture of iRT peptides (Biognosys) was spiked into all samples prior the measurement. Prior to LC separation, tryptic digests (approx. 2 μg of peptides) were online concentrated and desalted using trapping column (300 μm × 5 mm, μPrecolumn, 5 μm particles, Acclaim PepMap100 C18, Thermo Fisher Scientific; temperature of 40 °C). After washing of trapping column with 0.1% FA, the peptides were eluted (flow rate - 300 nl/min) from the trapping column onto an analytical column (Acclaim Pepmap100 C18, 3 μm particles, 75 μm × 500 mm; at a temperature of 40 °C, Thermo Fisher Scientific) by 109 min linear gradient program (5–37% of mobile phase B; mobile phase A: 0.1% FA in water; mobile phase B: 0.1% FA in 80% ACN). Equilibration of the trapping column and the analytical column was done prior to sample injection to sample loop. The analytical column outlet was directly connected to the Digital PicoView 550 (New Objective) ion source with sheath gas option and SilicaTip emitter (New Objective; FS360-20-15-N-20-C12) utilization. ABIRD was installed.

Data were acquired in a DIA mode. The survey scan covered m/z 350 to 1650 at resolution of 120,000 (at m/z 200) with AGC target value of 2 × 10^5^ and maximum injection time of 100 ms. HCD MS/MS (28% relative fragmentation energy) were acquired in the range of m/z 200 to 1800 at 30,000 resolution with a target value of 5 × 10^5^. The maximum injection time for MS/MS was 50 ms. Overlapping window patterns in m/z range from 400 to 1200 were used as isolation window placements – see supplemental File 1 for more details.

### DIA-LC-MS/MS Data Processing for Immunoprecipitation and Total Proteome Analysis

Quantitative analysis of the LC-MS/MS DIA data for immunoprecipitation and total proteome experiment was performed in Spectronaut 15.6 (Biognosys) software using the directDIA approach against human UniProt/SwissProt database (version 2021_03 downloaded on 2021-09-11, 20,371 sequences). Precursor Qvalue cutoff and experiment protein Qvalue cutoff were set to 0.01. For total proteome experiment, peptides identified with Qvalue <0.01 in at least 4 of 8 analyses were included (Qvalue percentile 0.5 setting). For immunoprecipitation, peptides identified with Qvalue <0.01 in at least 6 of 24 analyses were included (Qvalue percentile 0.25 setting). Fixed modifications were set to Carbamidomethyl (C); variable modifications were set to acetyl (Protein N-term) and oxidation (M). Other parameters were set as default: enzyme specificity trypsin/P allowing two missed cleavages, dynamic RT prediction and dynamic mass tolerances for precursor and fragment ions with correction factor 1, peptide identification required at least three fragment ions, and major and minor group quantities were based on mean peptide and mean precursor quantity, respectively. Differential abundance testing was performed using Student’s *t* test in Spectronaut 15.6; proteins with absolute Log2 Fold Change (|Log2FC|) > 0.58 and with q-value <0.05 were considered differentially abundant between sample groups. The visualization of total proteome results was performed in GraphPad software, version 9.3.1. The top 20 interaction partners of RELA from the immunoprecipitation experiment were visualized in Cytoscape, version 3.9.1.

### Gene Set Enrichment Analysis

Gene set enrichment analysis (GSEA) in GSEA Java desktop application (55) version 4.2.3 was conducted using the list of all quantified proteins from the immunoprecipitation experiment pre-ranked according to Log2 fold change to identify enriched pathways, with *a priori* defined pathways from Hallmark database. Minimal size of a gene set was adjusted to 2; otherwise default settings were used.

### AlphaPulldown Prediction of Protein Complexes

Protein/protein complexes were predicted by the AlphaFold-Multimer software (56, 57) incorporated into the AlphaPulldown (58) screening pipeline. Third version of AlphaFold-Multimer parameters was utilized. Following complexes were evaluated: KIF5B-SHTN1, KIF5B-PRMT5, PPP4R3A-SHTN1, and CSNK2A1-PPP4R3A. Predicted structures were evaluated based on their structural properties and scores. Mainly, we employed predicted alignment error (57), mpDockQ (59, 60), and PI_scores (61).

## Results

### NF-κB Inhibition Strongly Affects Protein–Protein Interactome in MCF-7 cells

**Fig. 1:**
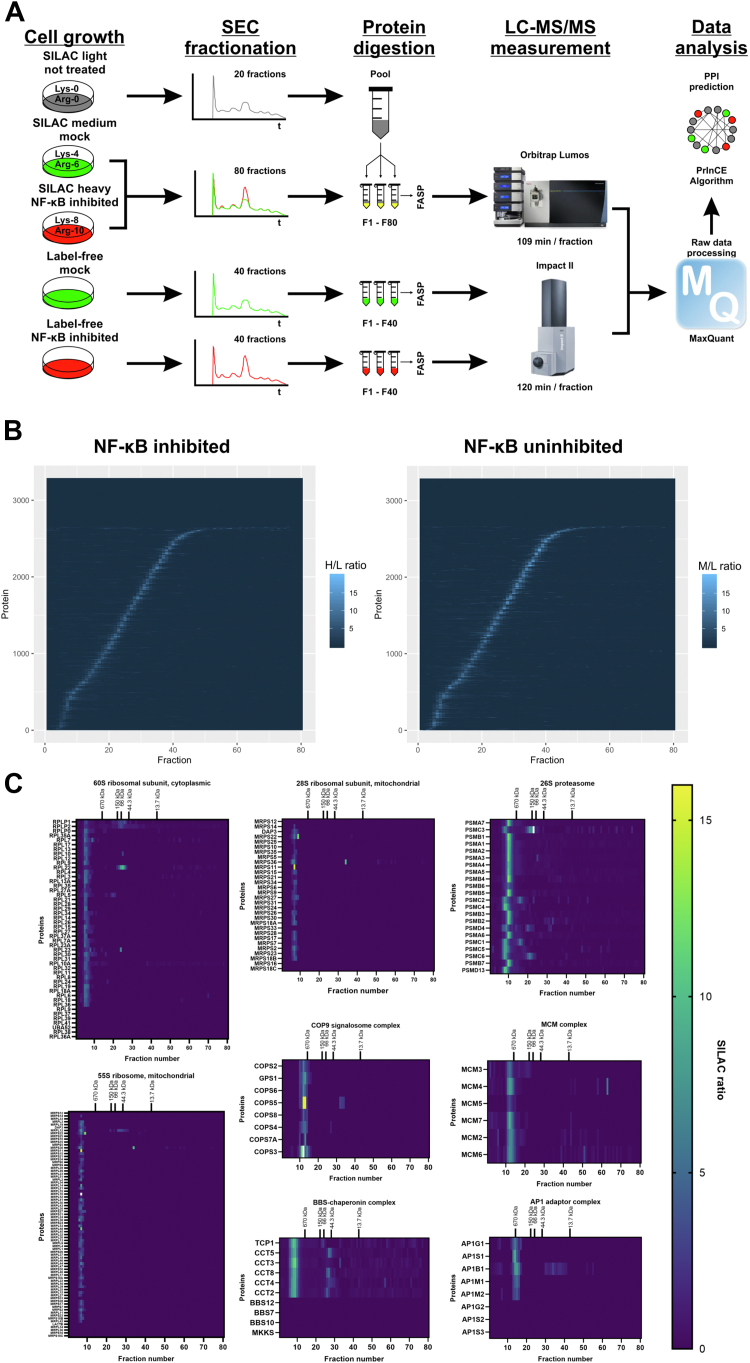
**Global mapping of protein interactome.***A*, overview of the SEC-PCP-SILAC interactomics experiment. *B*, heavy/light and medium/light SILAC ratios of proteins in NF-κB–inhibited and NF-κB–uninhibited cells of the SEC-PCP-SILAC replicate, respectively. *C*, SEC co-elution of proteins consisting known protein complexes in the CORUM database. SEC, size-exclusion chromatography; SILAC, stable isotope labeling by amino acids in cell culture; PCP, protein correlation profiling.

To compare protein interactome in MCF-7 breast cancer cell line with inhibited and uninhibited NF-κB pathway, we applied SEC-PCP-SILAC quantitative proteomics approach (19) (Fig. 1*A*). For our purpose, MCF-7 breast cancer cells labeled with heavy amino acids were transfected with plasmid encoding IκBα protein (NFKBIA) resistant to degradation (23) that inhibits transcriptional activity of NF-κB transcription factors and is also known as IκB-super-repressor. Control MCF-7 cells transfected with empty plasmid were labeled with medium isotopes. Presence of the FLAG-tagged IκBα fusion protein in cells transfected with IκB protein NFKBIA encoding plasmid was confirmed by SDS-PAGE and Western blotting with immunodetection (supplemental fig. s1*A* and Supplemental Methods). Heavy and medium labeled lysates were pooled and fractionated into 80 fractions using SEC. Light cell population lysate was fractionated separately, fractions were pooled, and the pool aliquots were distributed in heavy/medium fractions as internal standard. Resulting fractions were separately analyzed using LC-MS/MS (Fig. 1*A*). The SEC chromatograms for individual proteins were reconstructed based on the heavy/light and medium/light SILAC ratios for cell samples with inhibited and uninhibited NF-κB, respectively. The second biological replicate included label-free dataset (SEC-label-free) consisting of 40 SEC fractions from cells with inhibited NF-κB and of 40 SEC fractions from cells transfected with empty plasmid (Fig. 1*A*, supplemental Fig. S1*B*). MaxQuant data analysis led to identification of 43,642 peptides and 3308 protein groups across 80 SEC-PCP-SILAC fractions (FDR = 0.01, Fig. 1*B*, supplemental File 2) and of 337,823 peptides and 5460 protein groups across SEC-label-free fractions (FDR = 0.01, supplemental File 3).

**Fig. 2:**
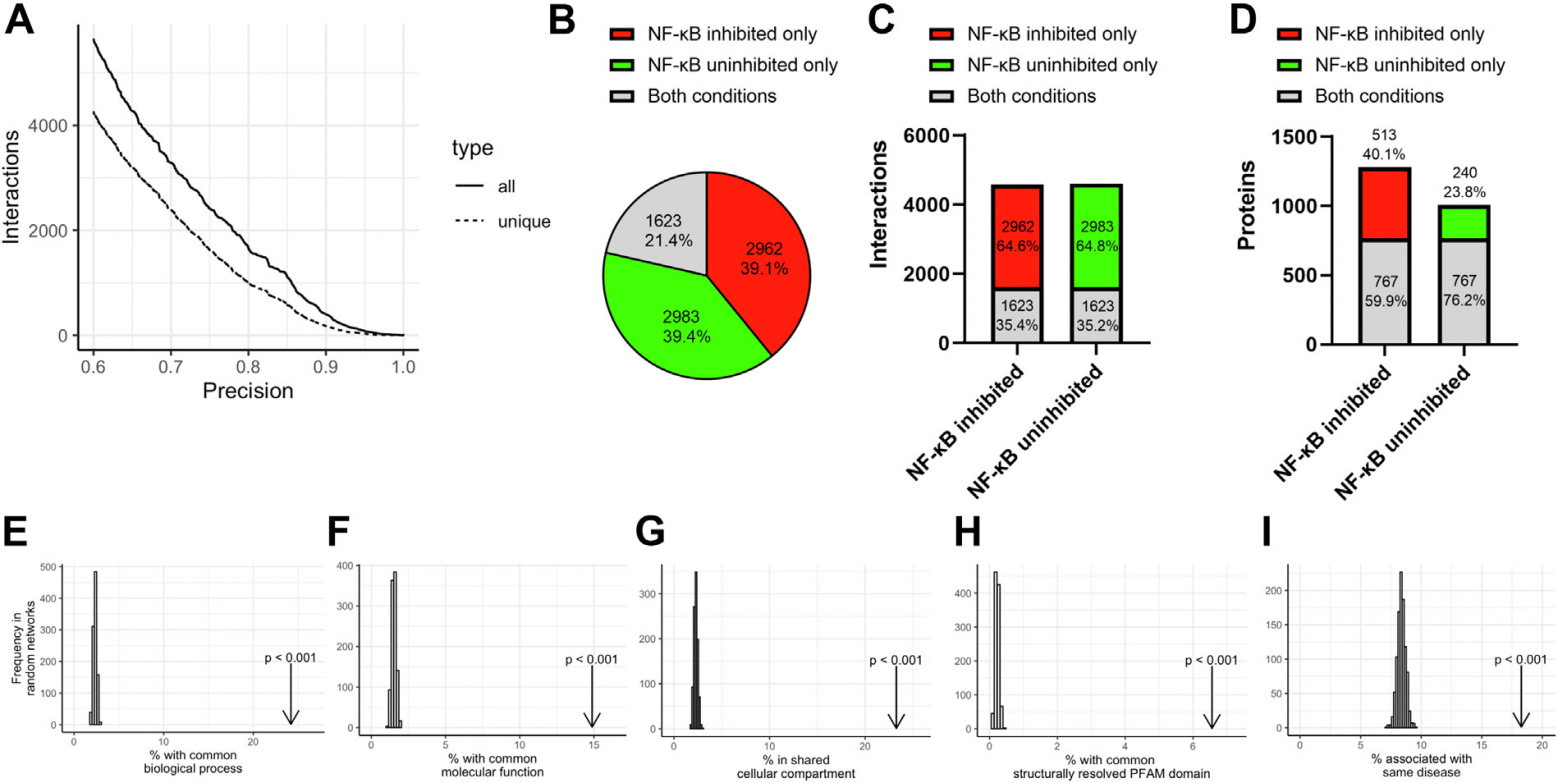
**Biological relevance of the mapped interactome.***A*, precision of protein–protein interactions in NF-κB–inhibited and NF-κB–uninhibited networks. *B*, percentage of unique interactions for NF-κB–inhibited and NF-κB–uninhibited networks and shared interactions in both conditions. *C*, ratio of unique and shared interactions in NF-κB–inhibited and NF-κB–uninhibited networks. *D*, ratio of unique and shared proteins in NF-κB–inhibited and NF-κB–uninhibited networks. Proportion of interacting protein pairs sharing at least one (*E*) biological process, (*F*) cellular compartment, or (*G*) molecular function GO term in the SEC-PCP-SILAC interactome. *H*, proportion of interacting protein pairs supported by a domain–domain interaction. *I*, proportion of interacting protein pairs implicated in the same disease. GO, gene ontology; SEC, size-exclusion chromatography; SILAC, stable isotope labeling by amino acids in cell culture; PCP, protein correlation profiling.

Subsequently, we applied the PrInCE algorithm (20) to calculate binary protein–protein interactions from our generated cofractionation datasets. The information from the CORUM protein complex database (29) was used to train the PrInCE classifier, determine TP and FP interactions and to calculate interaction precision. Our results show 7568 interactions with precision higher than 50% among 1520 protein groups, excluding known FP interactions (Fig. 2*A*, supplemental File 4). Of these, 1387 interactions were validated in CORUM database. These interactions involve co-eluting proteins forming complexes that were commonly observed in co-fractionation studies (30) such as ribosome, 26S proteasome, MCM, and COP9 complexes (Fig. 1*C*), confirming our SEC-PCP-SILAC experiment to reveal physical interactions within well-known protein complexes. In total, 2564 (33.9%) of our interactions detected by co-fractionation were validated in 15 protein–protein interaction databases and six published interactomics studies performed in various biological models using different methods (supplemental File 4), confirming their existence and suggesting biological role in breast cancer cells.

From the detected interactions, 4585 and 4606 binary protein–protein interactions occurred among 1280 and 1007 proteins in NF-κB–inhibited and NF-κB–uninhibited interaction networks, respectively (Fig. 2, *B*–*D*). From these, only 1623 interactions and 767 proteins were detected under both conditions (Fig. 2, *B*–*D*). These data suggest a strong effect of NF-κB inhibition on protein interactome since 78.5% of all identified interactions were detected exclusively only under one condition (Fig. 2*B*). Comparison of the whole NF-κB–inhibited and NF-κB–uninhibited interaction networks was visualized in supplemental Fig. S2.

Next, to demonstrate the biological relevance of our cofractionation results, we performed a permutation test to evaluate the involvement of interacting protein pairs in the same GO terms, including GO Biological Process, GO Molecular Function, and GO Cellular Compartment (Fig. 2, *E*–*G*). GO terms of all these three categories were statistically significantly shared between the interacting protein pairs (*p* < 0.001). Moreover, interacting proteins tended to share structurally resolved PFAM domains (*p* < 0.001, Fig. 2*H*). The permutation test also revealed association of interacting protein pairs with the same disease (*p* < 0.001, Fig. 2*I*). These results confirm co-elution of functionally related proteins.

### NF-κB Activity is Associated with Rearrangement of Protein Complexes Playing a Role in Immune Response and Cell Cycle

To characterize the effect of NF-κB pathway inhibition on MCF-7 protein interactome in relation to biological processes, we performed a pathway enrichment analysis based on comparison of differences between binary protein–protein interactions associated with functional GO categories in both NF-κB–inhibited and NF-κB–uninhibited networks and random network rewiring (21).

**Fig. 3:**
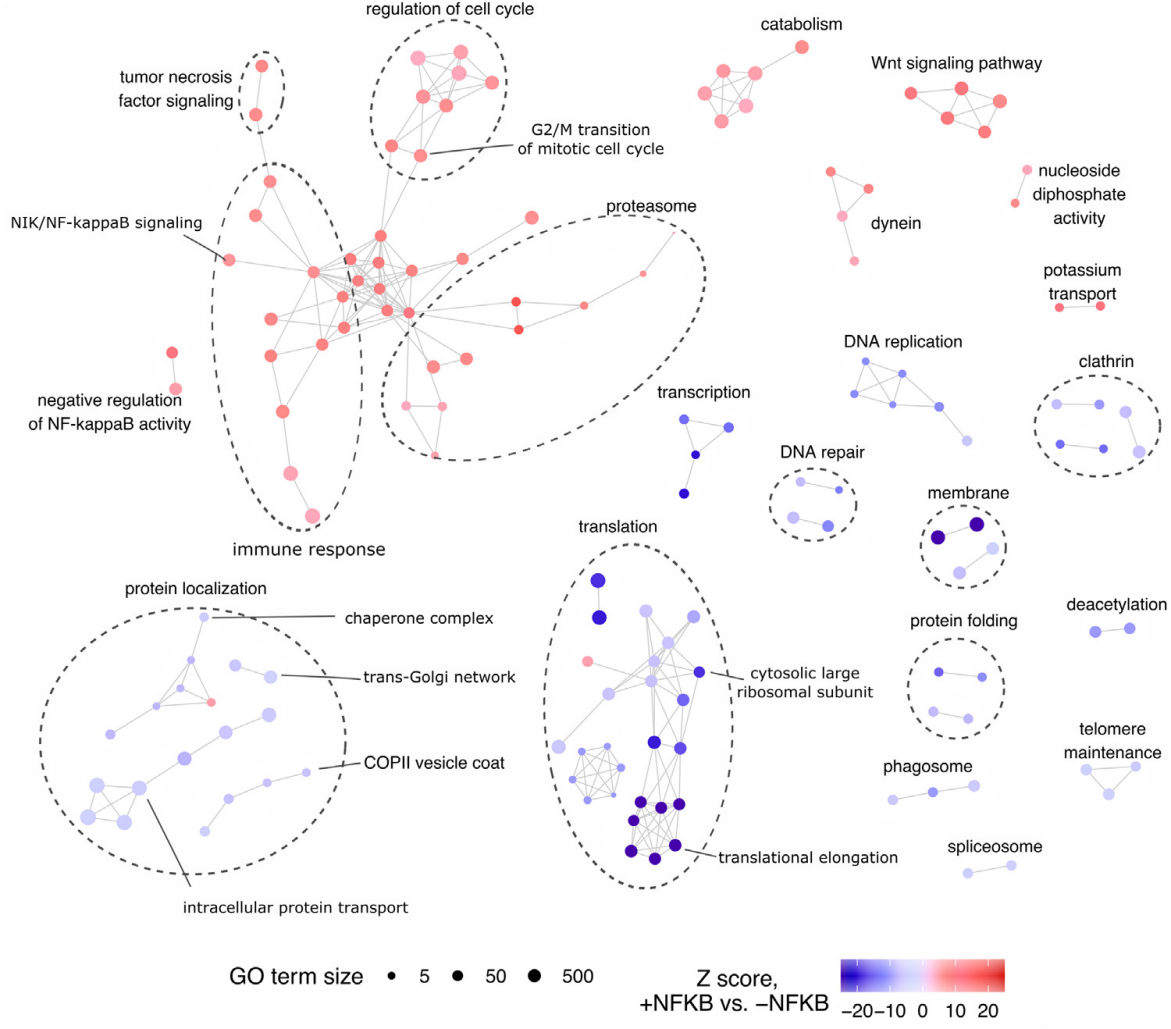
**Enrichment map of GO pathways from differential network analysis of NF-κB–inhibited and NF-κB–uninhibited interaction networks.** Nodes represent GO terms significantly negatively (*red*) or positively (*blue*) enriched in the NF-κB–inhibited interactome. Edges express the protein overlap between GO terms; Jaccard index cutoff was set to 0.33. GO, gene ontology.

**Fig. 4:**
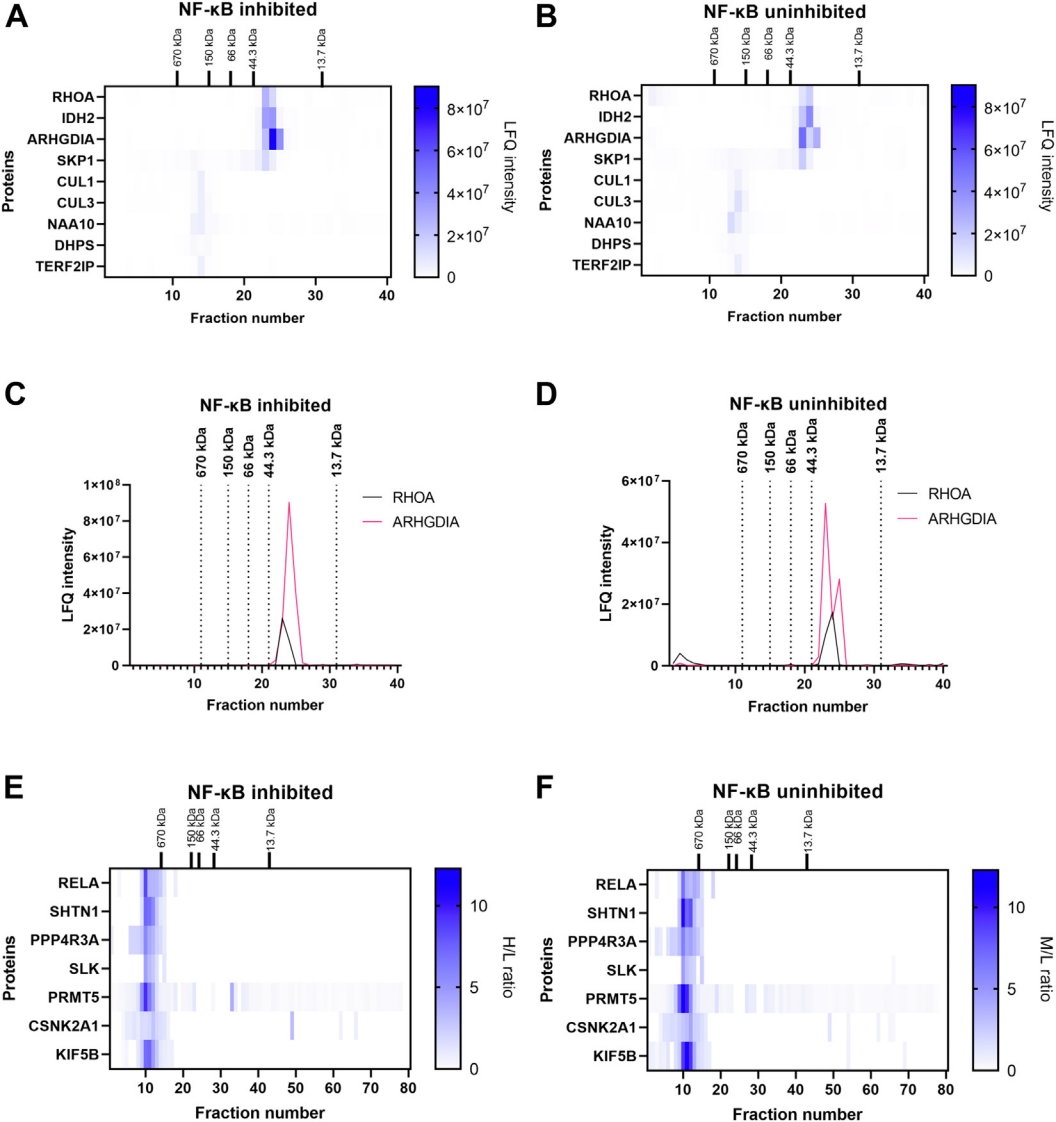
**Co-elution of proteins related to NF-κB pathway.** SEC co-elution of proteins from the “NIK/NF-kappaB signaling” pathway (*A*) from NF-κB–inhibited cells and (*B*) from NF-κB–uninhibited cells of the SEC-label-free replicate. SEC elution chromatograms of RHOA and ARHGDIA in (*C*) NF-κB–inhibited and (*D*) in NF-κB–uninhibited network of the SEC-label-free replicate. *E*, SEC co-elution of RELA with its protein interaction partners and their selected interactors in MCF-7 cells with inhibited NF-κB in the SEC-PCP-SILAC replicate. *F*, SEC co-elution of RELA with its protein interaction partners and their selected interactors in MCF-7 cells with uninhibited NF-κB in the SEC-PCP-SILAC replicate. SEC, size-exclusion chromatography; SILAC, stable isotope labeling by amino acids in cell culture; PCP, protein correlation profiling.

In general, 222 GO pathways were significantly enriched (q-value <0.05) in NF-κB–inhibited or NF-κB–uninhibited networks (supplemental File 5) and visualized as an enrichment map (Fig. 3). As the cell treatment influenced NF-κB pathway activity, the protein complexes involved in a pathway “NIK/NF-kappaB signaling” were significantly enriched. Apart from proteasomal subunits and the NF-κB transcription factor RELA, these reorganized complexes included transforming protein RHOA, ubiquitin ligases SKP1 and CUL1, and a transcription regulator TERF2IP (supplemental File 5). The RHOA protein co-eluted with its previously identified interaction partner IDH2 (Fig. 4, *A* and *B*), a known regulator of NF-κB pathway (62, 63) in fractions corresponding to molecular weight below 44 kDa. NF-κB inhibition was related to disruption of high molecular weight complexes including RHOA and ARHGDIA (Fig. 4, *A*–*D*), which is a negative regulator of Rho proteins (64) and a known RHOA interactor. In NF-κB–inhibited cells, CUL1 formed a complex with CUL3 reported by other studies (65, 66) and co-eluted with NAA10 and DHPS (Fig. 4, *A*–*B*), regulators of NF-κB pathway (67, 68). Based on these observations, we conclude that global mapping of protein complexes captures the changes induced by NF-κB inhibition in protein complexes linked to regulation of NF-κB pathway activity.

As the NF-κB plays a key role in regulating the immune response (1), we expected inhibition of NF-κB pathway to influence interactions of proteins involved in related biological processes. Indeed, pathways associated with innate immune response, inflammation, antigen presentation, as well as IL-1 pathway and leukocyte receptors were significantly enriched among the interactions decreased by NF-κB inhibition. Nevertheless, we asked whether the interactome changes are linked also to other cellular functions that could offer an insight into the role of NF-κB in cancer progression. Interestingly, interactions decreased by NF-κB inhibition were also enriched for pathways connected to cell cycle regulation and Wnt signaling pathway, indicating NF-κB inhibition to induce changes in protein complexes that play a role in key mechanisms of tumorigenesis. The NF-κB inhibition affected also other cellular processes such as pathways of DNA replication, transcription, translation, and protein localization and folding that were enriched in the interaction network of NF-κB–uninhibited cells. These results demonstrate association of NF-κB pathway activity to a broad range of cellular processes via protein–protein interactions, including mechanisms associated with tumorigenesis.

### RELA-Interacting Proteins Co-complex with NF-κB Activator PRMT5

Among the members of the NF-κB transcription factor family, interactions of RELA were detected in our co-fractionation data (supplemental Fig. S3). To our knowledge, 31 proteins produced by known NF-κB target genes were present in our interaction data (supplemental File 6), including CTSB, HSP90AA1, IGFBP2, and TRAF2. Total of 111 proteins present in our SEC-PCP-SILAC interaction networks were the known interaction partners of RELA from protein interaction databases (supplemental File 7). Although other NF-κB factors were not detected in the SEC-PCP-SILAC dataset and are thus missing in the interaction networks, co-elution of four NF-κB factors and three inhibitory IκB proteins, including the NFKBIA protein, was observed in the SEC-label-free experiment, confirming occurrence of NF-κB factors and IκB inhibitory proteins in the complexes (for SEC chromatograms, representative MS/MS spectra, and extracted ion chromatograms of NFKBIA, please see supplemental Fig. S4). According to our SEC-PCP-SILAC results, RELA co-eluted with SHTN1, KIF5B, and SLK proteins in cells with inhibited NF-κB pathway (Fig. 4*E*, supplemental Fig. S3); on the other hand, RELA co-complexed with PPP4R3A in NF-κB–uninhibited cells (Fig. 4*F*, supplemental Fig. S3). From the known interaction partners of RELA in protein interaction databases, PRMT5 interacted with SHTN1 and KIF5B in our results. Interactions SHTN1-PRMT5 and KIF5B-PRMT5 were further validated in other studies (supplemental File 4), suggesting a possible unexplored role of SHTN1 and KIF5B in the physical interplay between RELA and PRMT5. Moreover, PPP4R3A co-eluted with CSNK2A1, known RELA binding partner (69) and activator (70, 71, 72); this potential interaction was not observed previously. Our results suggest identification of novel RELA interaction partners that co-elute with known interactors of RELA.

Co-elution of NF-κB factors and IκB proteins as well as co-elution of RELA, PPP4R3A, SHTN1, KIF5B, SLK, PRMT5, and CSNK2A1 proteins was confirmed in our additional independent dataset consisting of 34 SEC fractions of native protein complexes extracted from unlabeled WT MCF-7 cells (for SEC chromatograms, see supplemental Fig. S5, for complete protein group list see supplemental File 8, and for methods please see Supplemental Methods).

### AlphaPulldown Predicts Complexes of Co-eluting Proteins

To confirm the ability of the key discussed co-eluting proteins to form complexes, we employed the AlphaPulldown (58) methodology to predict interactions detected in our cofractionation experiment among KIF5B-SHTN1, KIF5B-PRMT5, PPP4R3A-SHTN1, and CSNK2A1-PPP4R3A protein pairs (for the details of analysis input please see supplemental Table S1). Complexes of RELA protein could not be evaluated due to their interaction with DNA that prevents from the prediction by AlphaFold-Multimer. All of these interactions had at least one predicted alignment error between interface residues of the protein chains lower than the thresholds of 5 and 10 Å (please see Table 1 for resulting interaction scores and supplemental Table S2 for detailed characterization). Three complexes had a positive value of PI-score, confirming quality of the prediction. The AlphaPulldown pipeline predicted the interaction between KIF5B/SHTN1 proteins as the most probable. The other interactions KIF5B/PRMT5, PPP4R3A/SHTN1, and CSNK2A1/PPP4R3A were also predicted. The AlphaPulldown prediction thus confirmed the probability of complex formation between proteins that co-eluted with NF-κB activators.

**Table 1:** Complexes of the co-eluting proteins predicted by the AlphaPulldown methodology

Protein A	Protein B	PAE cutoff		SC	Esolv	Sint	PI-score	iPTM+PTM	mpDockQ /pDockQ
		5 Å	10 Å						
CSNK2A1	PPP4R3A		x	0.25	4.7	2192.7	−0.88	0.29	0.19
PPP4R3A	SHTN1		x	0.17	−16.8	3041.0	0.62	0.25	0.11
KIF5B	PRMT5		x	0.05	−20.7	4705.0	0.83	0.33	0.70
KIF5B	SHTN1	x	x	0.33	−103.0	6851.9	0.92	0.39	0.46

iPTM+pTM scores are reported by AlphaFold-Multimer. mpDockQ/pDockQ (higher is better) is calculated using the formula given by Bryant et al (59, 60). PI-score (higher is better) and the rest of the columns are reported by the PI-score pipeline (61). SC: geometric shape complementarity of protein/protein interfaces. SC ranges between 0 and 1, with sc = 1 being two proteins mesh precisely. Esolv: interface solvation energy. Sint: interface surface area inaccessible to solvent upon the interface formation [Å^2^].

### Immunoprecipitation Reveals Inhibition of NF-κB Pathway via Modulating RELA Interactions with NF-κB Activators

**Fig. 5:**
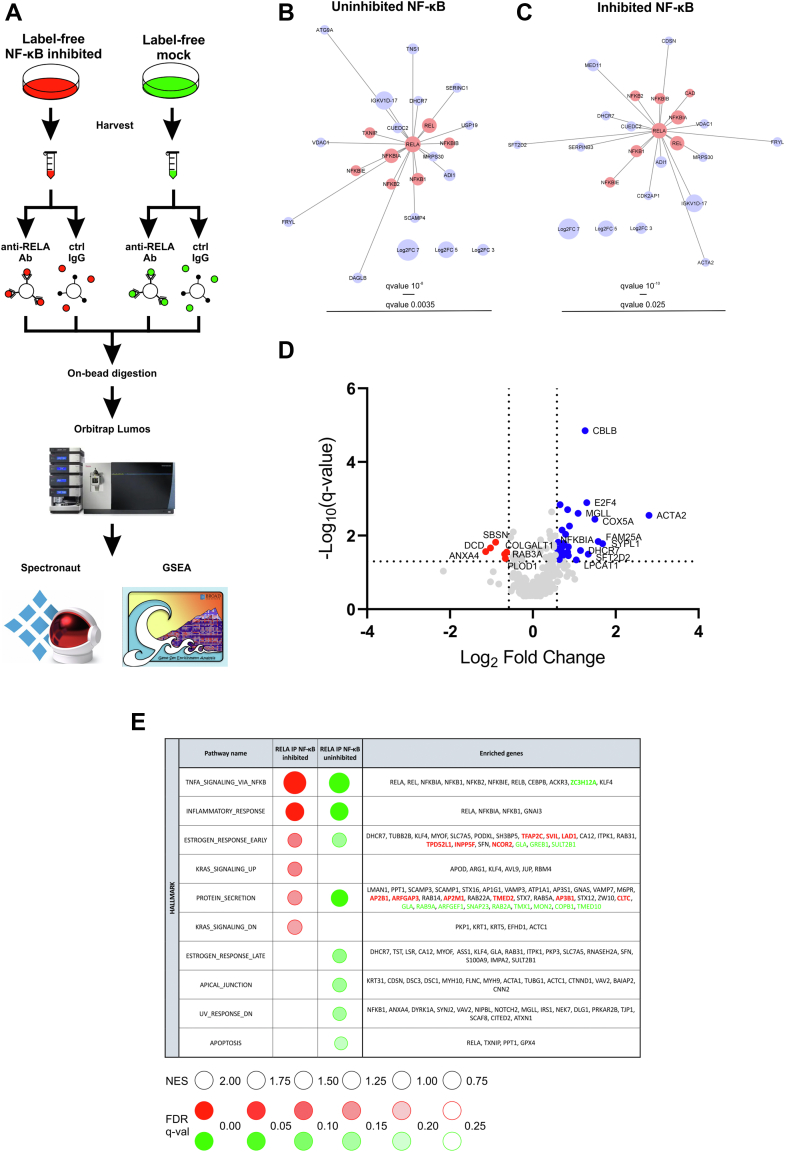
**Identification of RELA-interacting proteins using immunoprecipitation.***A*, overview of the RELA immunoprecipitation experiment. *B*, TOP 20 interaction partners of RELA in NF-κB–uninhibited cells. *C*, TOP 20 interaction partners of RELA in NF-κB–inhibited cells. Proteins in *red* are known interaction partners of RELA from protein interaction databases. *D*, volcano plot of protein interaction partners of RELA from the immunoprecipitation experiment comparing protein level abundances in immunoprecipitates from cells with inhibited and uninhibited NF-κB pathway. Horizontal line displays the statistical significance cutoff (q-value < 0.05). Vertical lines show the Log2 Fold Change >0.58 threshold for the upregulated proteins (in *blue*) and Log2 Fold Change < −0.58 threshold for the downregulated proteins (in *red*). *E*, positively enriched Hallmark pathways in GSEA analysis of RELA immunoprecipitation results. The genes in *red* were enriched in NF-κB–inhibited cells only. The genes in *green* were enriched in NF-κB–uninhibited cells only.

To further explore the interactome of RELA and to evaluate its status after NF-κB inhibition, we implemented an immunoprecipitation experiment with NF-κB–inhibited and NF-κB–uninhibited MCF-7 cells. Monoclonal anti-RELA antibody binding to residues surrounding Glu498 of RELA protein or control IgG was used; interacting proteins were quantified with LC-DIA-MS/MS in six biological replicates per condition (Fig. 5*A*, supplemental Fig. S6). In total, 4184 proteins were identified (FDR = 0.01, supplemental File 9). In NF-κB–uninhibited cells, 191 proteins were identified as interaction partners of RELA (Log2FC > 0.58, q-value<0.05, supplemental File 9). The TOP20 interacting proteins of RELA (Log2FC = 4.71, q-value = 1.91E-08) included NF-κB factors REL, NFKB1, and NFKB2, as well as IκB inhibitory proteins NFKBIA, NFKBIB, and NFKBIE (Fig. 5*B*). NF-κB factor RELB was present among the interacting proteins as well. Highly abundant interacting proteins included ADI1 involved in methionine salvage and polyamine synthesis pathways (73), TXNIP, a regulator of NF-κB (74), and TNS1, protein related to cell adhesion and migration (75). In total, 17 of the RELA-interacting proteins were the interactors known from the interaction databases (supplemental File 9), including TXNIP, CAD, a negative regulator of NF-κB (76), ubiquitin conjugating enzyme UBE2E1, and DCD, a positive regulator of RELA (77). In conclusion, our immunoprecipitation experiment captured interactions of RELA with other NF-κB factors and regulators of NF-κB pathway and extended the known interactome of RELA in breast cancer cells.

In NF-κB–inhibited cells, we identified 150 RELA interaction partners (Log2FC > 0.58, q-value < 0.05, Fig. 5*C*, supplemental File 9). The NF-κB inhibition resulted in statistically significant (q-value < 0.05) upregulation (Log2FC > 0.58) and downregulation (Log2FC < −0.58) of 31 and 6 RELA-interacting proteins, respectively (Table 2, Fig. 5*D*). As expected, transfection of MCF-7 cells with plasmid encoding IκB protein NFKBIA led to increased binding of NFKBIA to RELA (Log2FC = 0.61, q-value = 0.015, Table 2). Nevertheless, another IκB protein, NFKBIB, had elevated levels after NF-κB inhibition in RELA immunoprecipitate (Log2FC = 0.84, q-value = 0.028, Table 2). The upregulated proteins included E3 ubiquitin protein ligase CBLB and regulator of transcription BACH1. On the other hand, NF-κB inhibition decreased interactions of RELA with ANXA4 that formed a feedback regulatory loop with RELA (78), DCD, and SBSN, a potential downstream target of NF-κB pathway (79). Moreover, we observed decreased levels of RELA-interacting protein PLOD1 known as a positive regulator of NF-κB activity (80) in inhibited cells and its known interaction partner COLGALT1. Inhibition of NF-κB thus affected interactions of RELA with proteins involved in the regulation of NF-κB pathway.

**Table 2:** Overview of the RELA-interacting proteins with statistically significantly changed abundance in RELA immunoprecipitates after NF-κB inhibition (|Log2FC| > 0.58, q-value < 0.05)

UniProt Id	Gene	Protein description	Protein name	Inhibited RELA versus inhibited ctrl		Uninhibited RELA versus uninhibited ctrl		Inhibited RELA versus uninhibited RELA	
				Log2FC	q-value	Log2FC	q-value	Log2FC	q-value
P09525	ANXA4	Annexin A4	ANXA4_HUMAN	0.13	0.277	**1.08**	**0.028**	−1.14	0.027
P81605	DCD	Dermcidin	DCD_HUMAN	−0.53	0.107	**0.77**	**0.047**	−1.03	0.022
Q6UWP8	SBSN	Suprabasin	SBSN_HUMAN	−0.40	0.088	**0.70**	**0.021**	−0.90	0.015
P20336	RAB3A	Ras-related protein Rab-3A	RAB3A_HUMAN	−0.04	0.338	**0.75**	**0.035**	−0.68	0.032
Q02809	PLOD1	Procollagen-lysine,2-oxoglutarate 5-dioxygenase 1	PLOD1_HUMAN	0.27	0.326	**0.65**	**0.019**	−0.67	0.042
Q8NBJ5	COLGALT1	Procollagen galactosyltransferase 1	GT251_HUMAN	0.36	0.051	**0.64**	**0.019**	−0.63	0.028
Q7Z5P9	MUC19	Mucin-19	MUC19_HUMAN	**1.06**	**0.003**	0.54	4.22E-04	0.59	0.023
Q9NZM1	MYOF	Myoferlin	MYOF_HUMAN	0.55	0.104	**0.71**	**0.001**	0.60	0.034
P25963	NFKBIA	NF-kappa-B inhibitor alpha	IKBA_HUMAN	**4.00**	**5.96E-09**	**4.13**	**4.48E-08**	0.61	0.015
Q8IWE2	FAM114A1	Protein NOXP20	NXP20_HUMAN	**0.79**	**0.021**	0.16	0.143	0.64	0.045
Q13510	ASAH1	Acid ceramidase	ASAH1_HUMAN	0.56	0.044	**1.02**	**7.30E-06**	0.66	0.001
Q9NZD8	SPG21	Maspardin	SPG21_HUMAN	0.40	0.178	**0.62**	**0.010**	0.66	0.024
P51809	VAMP7	Vesicle-associated membrane protein 7	VAMP7_HUMAN	0.47	0.216	**1.06**	**0.002**	0.69	0.021
P49257	LMAN1	Protein ERGIC-53	LMAN1_HUMAN	**1.05**	**0.045**	0.45	0.016	0.69	0.021
O14867	BACH1	Transcription regulator protein BACH1	BACH1_HUMAN	**0.77**	**0.008**	0.47	0.026	0.70	0.020
O00625	PIR	Pirin	PIR_HUMAN	0.57	0.066	**0.87**	**2.00E-06**	0.70	0.028
P62834	RAP1A	Ras-related protein Rap-1A	RAP1A_HUMAN	**0.60**	**0.010**	**0.87**	**1.91E-07**	0.70	0.007
O00264	PGRMC1	Membrane-associated progesterone receptor component 1	PGRC1_HUMAN	**0.87**	**0.032**	**1.43**	**1.66E-04**	0.72	0.029
P50542	PEX5	Peroxisomal targeting signal 1 receptor	PEX5_HUMAN	**0.94**	**0.023**	0.47	0.009	0.73	0.034
Q9H4L7	SMARCAD1	SWI/SNF-related matrix-associated actin-dependent regulator of chromatin subfamily A containing DEAD/H box 1	SMRCD_HUMAN	**1.55**	**0.003**	−0.39	0.179	0.74	0.050
P35611	ADD1	Alpha-adducin	ADDA_HUMAN	**0.65**	**0.011**	0.42	0.002	0.75	0.016
Q9UNX3	RPL26L1	60S ribosomal protein L26-like 1	RL26L_HUMAN	0.23	0.406	**1.02**	**6.47E-07**	0.79	0.009
Q15653	NFKBIB	NF-kappa-B inhibitor beta	IKBB_HUMAN	**3.68**	**9.80E-07**	**2.89**	**2.68E-06**	0.84	0.028
Q6NXT1	ANKRD54	Ankyrin repeat domain-containing protein 54	ANR54_HUMAN	**0.71**	**0.003**	0.50	**9.91E-06**	0.84	0.002
Q9Y3B4	SF3B6	Splicing factor 3B subunit 6	SF3B6_HUMAN	0.55	0.096	**1.08**	**0.001**	0.86	0.035
Q9UKD2	MRTO4	mRNA turnover protein 4 homolog	MRT4_HUMAN	**1.33**	**0.004**	**0.94**	**3.71E-04**	0.86	0.020
L0R8F8	MIEF1	MIEF1 upstream ORF protein	MIDUO_HUMAN	0.56	0.102	**0.79**	**5.61E-05**	0.88	0.005
Q8NF37	LPCAT1	Lysophosphatidylcholine acyltransferase 1	PCAT1_HUMAN	0.26	0.343	**0.77**	**0.047**	1.05	0.046
Q99685	MGLL	Monoglyceride lipase	MGLL_HUMAN	**0.87**	**0.024**	0.31	0.257	1.09	0.002
Q9UBM7	DHCR7	7-dehydrocholesterol reductase	DHCR7_HUMAN	**2.97**	**4.19E-04**	**2.13**	**2.28E-05**	1.15	0.025
Q13191	CBLB	E3 ubiquitin-protein ligase CBL-B	CBLB_HUMAN	**0.91**	**0.007**	−0.27	0.005	1.26	1.41E-05
Q16254	E2F4	Transcription factor E2F4	E2F4_HUMAN	**0.77**	**0.027**	0.41	0.318	1.30	0.001
O95562	SFT2D2	Vesicle transport protein SFT2B	SFT2B_HUMAN	**1.69**	**0.026**	**1.52**	**0.007**	1.34	0.032
P20674	COX5A	Cytochrome c oxidase subunit 5A, mitochondrial	COX5A_HUMAN	**0.73**	**0.043**	0.32	0.001	1.50	0.004
B3EWG3	FAM25A	Protein FAM25A	FM25A_HUMAN	**1.16**	**0.030**	0.46	4.57E-04	1.57	0.014
Q16563	SYPL1	Synaptophysin-like protein 1	SYPL1_HUMAN	0.46	0.366	**1.43**	**6.30E-05**	1.68	0.017
P62736	ACTA2	Actin, aortic smooth muscle	ACTA_HUMAN	**1.75**	**0.025**	−0.05	0.358	2.80	0.003

Bold values indicate potential RELA interactors (Log2FC >0.58 and q-value <0.05 in the inhibited RELA *versus* inhibited ctrl or uninhibited RELA *versus* uninhibited ctrl comparison).

GSEA results of the complete lists of proteins quantified in RELA immunoprecipitates revealed enrichment of eight and six HALLMARK pathways among RELA-interacting proteins from NF-κB–uninhibited and NF-κB–inhibited cells, respectively (NES >1, FDR q-value < 0.25, Fig. 5*E*, supplemental File 9). This analysis highlighted the strongest interactions of RELA with other NF-κB factors that participated in TNFA_SIGNALING_VIA_NFKB and INFLAMMATORY_RESPONSE pathways; however, other proteins, such as atypical chemokine receptor ACKR3, endoribonuclease ZC3H12A, and guanine nucleotide-binding protein GNAI3, were enriched in these pathways as well. The interaction partners of RELA were also associated with hormonal response in ESTROGEN_RESPONSE_EARLY and ESTROGEN_RESPONSE_LATE pathways, involving reductase DHCR7, transcription factor KLF4, and lipoprotein receptor LSR. The pathways affected by NF-κB inhibition included pathways associated with KRAS as well as APICAL_JUNCTION and APOPTOSIS pathways. These results indicate that RELA interacts with proteins linked to NF-κB signaling, inflammation, and other processes, including estrogen response.

### Interactome Rearrangement by NF-κB inhibition is not Driven by Changes in Protein Abundance

**Fig. 6:**
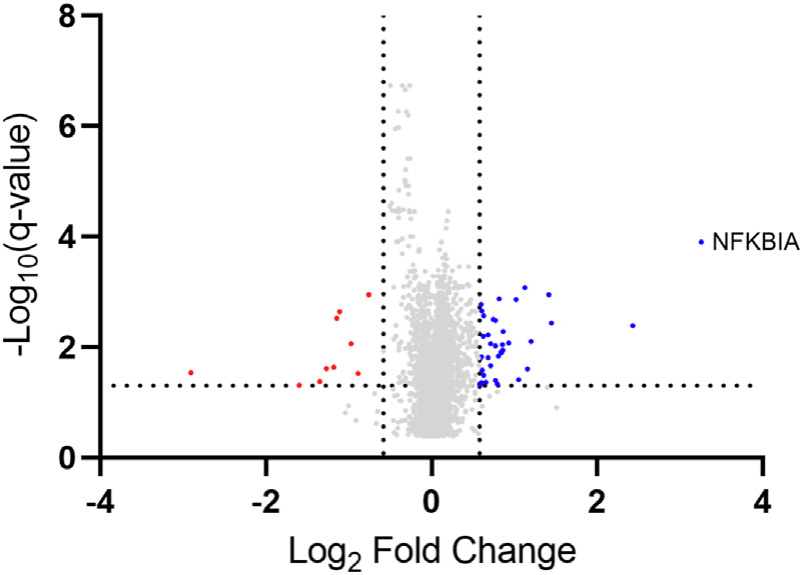
**Total proteome experiment.** Volcano plot of protein level changes in MCF-7 cells with inhibited NF-κB pathway. Horizontal line displays the statistical significance cutoff (q-value < 0.05). Vertical lines show the Log2 Fold Change >0.58 threshold for the upregulated proteins (in *blue*) and Log2 Fold Change < −0.58 threshold for the downregulated proteins (in *red*).

To confirm that the rearrangements of protein complexes are not changed simply by changes in protein abundances resulting from altered gene expression, we analyzed the effect of NF-κB inhibition on the MCF-7 total proteome. We analyzed aliquots of transfected MCF-7 cells of the SEC-label-free replicate with plasmid encoding IκB protein NFKBIA or with empty plasmid and quantified protein abundance by LC-MS/MS analysis in DIA mode. A total of 6152 protein groups were identified (FDR = 0.01, supplemental File 10). From these, only 37 and 10 proteins were significantly (q-value < 0.05) upregulated (Log2FC > 0.58) and downregulated (Log2FC < −0.58), respectively, with the NFKBIA protein as the most upregulated in the cells with inhibited NF-κB pathway (Fig. 6) which internally validates our experimental design (for extracted ion current chromatograms of NFKBIA precursors, please see supplemental Fig. S7). Comparison of co-fractionation interactome data and the total proteome data provided the evidence that any of the changes in interactions given by NF-κB inhibition in SEC-PCP-SILAC experiment were not caused simply by changes in protein levels/expression (supplemental File 4, no protein with |Log2FC| > 0.58 and q-value < 0.05 in Total proteome analysis columns). In immunoprecipitation experiment, only a single interaction partner of RELA, KRAS protein, was found co-expressed with NFKBIA in total proteome experiment (supplemental File 9). In conclusion, these data confirm that the cellular mechanisms associated with NF-κB activity in our model were generally regulated through rearrangement of protein interactions.

## Discussion

In this study, we applied the SEC-PCP-SILAC approach to identify protein interactions specific for MCF-7 cell interactome under innate NF-κB transcription activity and to identify changes in the composition of protein complexes associated with NF-κB inhibition. This is a critical progress over previous studies which either described the interactome in MCF-7 cells without any modulation (46) or focused on interaction network of NF-κB pathway using targeted methods, not reflecting the complex composition on proteome-scale (81, 82). In our interaction map, modulation of NF-κB was related to extensive changes in MCF-7 protein interactome involving protein complexes that participate in various cellular processes. As the NF-κB is best known as a key regulator of inflammation and immune response (83, 84, 85), we indeed detected rearrangement of protein complexes involved in NF-κB signaling, innate immune response, antigen presentation, and IL-1 pathways. However, other studies demonstrated NF-κB pathway to support cancer development and progression, including lymph node metastasis of luminal A breast tumors (16, 17), as it contributes to number of other cellular processes and promotes cellular growth and proliferation (86, 87, 88). We demonstrated the effects of NF-κB activity on composition of protein complexes associated with regulation of cell cycle and Wnt pathway that represents an important therapeutic target in breast cancer (89, 90). Our current interaction network offers detailed insight into association of key cellular processes with NF-κB in MCF-7 breast cancer model and maps rearrangement of protein complexes involved in pro-tumorigenic pathways.

Our interaction dataset includes NF-κB transcription factor RELA that co-eluted with SHTN1, KIF5B, PPP4R3A, and SLK proteins. SHTN1, a protein co-eluting with RELA in our protein interaction data after NF-κB inhibition, is a regulator of phosphoinositide 3-kinase activity, axon outgrowth (91), cytoskeletal organization, and cell adhesion (92). SHTN1 was previously identified as a candidate interactor of IKBKG, a subunit of IκB kinase (93). KIF5B contributes to lysosome membrane and mitochondria transportation (94, 95) and central spindle organization (96). In our SEC-PCP-SILAC data, SHTN1 and KIF5B co-eluted with PRMT5 protein; the KIF5B-PRMT5 complex was further validated by AlphaPulldown. PRMT5, as member of the protein arginine methyltransferase superfamily, is generally involved in the dimethylation of histone and non-histone proteins (97) and interacts with several transcription factors including RELA, as well as with splicing and transcription elongation factors (98). Due to these interactions as well as its increased expression in tumor cells, PRMT5 was linked to cancer proliferation, migration, epithelial to mesenchymal transition, invasion, and metastasis (98) and was suggested as potential therapeutic anti-cancer target (99, 100). PRMT5 was also found to play a role in cancer cell growth (101). Interestingly, PRMT5 dimethylated R30 of RELA which led to its increased transcription activity (102). Our study confirms at several levels that SHTN1 and KIF5B interact with both PRMT5 and RELA in luminal A breast cancer cells.

The immunoprecipitation experiment performed with anti-RELA antibody further expanded the known protein interactome of RELA in MCF-7 breast cancer cell line. Apart from the complexes of RELA with other NF-κB factors, IκB proteins, and several known regulators of NF-κB pathway, we observed RELA to interact with proteins involved in estrogen response. Other studies revealed a crosstalk between NF-κB pathway and estrogen receptor (103) that can be either positive (104) or negative (105, 106, 107, 108). Our results thus uncovered new mechanisms in the relationship between NF-κB and estrogen pathways on the level of protein–protein interactions. We moreover detected increased levels of proteins from KRAS signaling pathway in RELA immunoprecipitates from the cells with inhibited NF-κB. This could be caused by the increased expression of NF-κB regulator KRAS (109) as we observed elevated KRAS protein levels in total proteome after NF-κB inhibition. Further, the inhibition of NF-κB affected RELA interactors involved in cell adhesion and apoptosis. As NF-κB activation is associated with cellular adhesion (110) and affects apoptosis (111), these pathways could represent pro-tumorigenic mechanisms related to NF-κB in breast cancer. Immunoprecipitation experiment also revealed decreased binding of ANXA4, DCD, and PLOD1 to RELA after NF-κB inhibition. ANXA4 was found to bind NFKB1/p50 protein and to activate NF-κB signaling pathway in ovarian carcinoma (112). In gallbladder carcinoma, knockdown of ANXA4 resulted in decreased transcription activity of RELA and lower expression of NF-κB target genes; moreover, inhibition of NF-κB was associated with suppressed ANXA4 expression (78). ANXA4 was overexpressed in breast cancer tissues (113) and its overexpression stimulated MCF-7 cell migration (114). The decreased abundance of ANXA4 in RELA immunoprecipitate after NF-κB inhibition in our study suggests that ANXA4 interplays with NF-κB also in the breast cancer and could promote metastatic behavior of breast tumors via NF-κB pathway. DCD expression in breast tumors was associated with increased tumor size and presence of lymph node metastases (115), enhanced proliferation of 21NT breast cancer cell line (115), and migration of MCF-7 cells (116). DCD was found to promote phosphorylation and thus degradation of NFKBIA in keratinocytes, leading to increased NF-κB activity (77). PLOD1 expression was increased in breast tumors compared to normal breast tissues, promoted lymph node, and lung metastasis (117), and its expression in breast cancer cells was induced by hypoxia (117). PLOD1 interacted with NFKBIA protein and activated the NF-κB pathway (80), probably through NFKBIA phosphorylation and degradation (80). Briefly, our immunoprecipitation results link the NF-κB inhibition with decreased binding of NF-κB activators ANXA4, DCD, and PLOD1 to transcription factor RELA.

As every study, also this one has its limitations. As the proteins identified here as interacting occur in the same protein complexes, some of the detected complex forming interactions may not be always direct but also indirect. Our interaction network is based on protein co-elution detected by two SEC-PCP approaches that were confirmed by a third independent SEC-LC-MS/MS dataset for all interactions discussed in the manuscript text. Part of the detected interactions were also present in the interaction databases and other interactomics studies, nonetheless, the interactions that were not validated would require further confirmation by an independent method before building new biological hypotheses. Moreover, as SEC-PCP-SILAC maps rearrangement of thousands of protein complexes, some known protein complexes can be missing in the global interaction network due to limitations in sensitivity. We also performed prediction of possible protein complexes using very recent AlphaPulldown approach between the key co-eluting protein pairs. Although AlphaPulldown confirmed some of the binary protein interactions, we did not confirm any complex of RELA, which is probably due to i) possible RELA interactions with DNA, ii) structures of interacting proteins containing disordered regions that impede the prediction, iii) protein oligomerization and folding of multiprotein complexes. In our approach, we further complement the co-fractionation interactions with identification of interaction partners of RELA protein using immunoprecipitation that is based on protein enrichment together with its interacting proteins. Immunoprecipitation thus represents a complementary method to SEC-PCP-SILAC for sensitive characterization of interactome of a selected protein. Nevertheless, immunoprecipitation identifies only strong interactions in the enriched complexes, as the weak complexes are disrupted during the washing steps, and the antibody binding the region near C-terminus of RELA modulates the binding sites of the RELA protein, targeting different portion of detectable interactome compared to PCP-SILAC. Furthermore, the SEC-PCP-SILAC workflow includes SILAC labeling which is not compatible with every cell line. The MCF-7 cell line used in our work is estrogen dependent and it was rather difficult to grow it in media containing dialyzed FBS that is essential for proper SILAC labeling (118). Although we optimized the SILAC growth media composition, the growth of MCF-7 cells was relatively slow, which resulted in limited amount of protein material used in the experiment that affected the number of protein IDs compared to label-free approach. As RELA and its binding partners are of low protein abundance, the quantification of these proteins using SEC-PCP-SILAC is limited by the dynamic range of protein abundance and amount of protein material available in the SEC fractions. However, the requirement of the SILAC labeling can be omitted by the use of label-free approach such as SEC-DIA/SWATH that has been recently shown to have a great potential in global interactome mapping (119). The absence of the SILAC labeling is, on the other side, related to significantly longer instrument acquisition time (119). In the SEC-PCP-SILAC workflow, use of reversed labeling could further improve the quantification reliability. Next, NF-κB stimulation could lead to detection of additional interactome changes that were not captured in our study based on NF-κB inhibition. On the other hand, some of the interactome changes observed in our results may be caused not only by the direct inhibition of NF-κB signaling but also by secondary or tertiary signaling events or adaptive effects upon inhibition of NF-κB. Finally, mapping of protein complexes associated with NF-κB in additional cell lines would be beneficial to validate the detected protein–protein interactions in more breast cancer models.

In conclusion, in this study, we constructed a proteome-scale interaction network in MCF-7 breast cancer cell line using SEC-PCP-SILAC method and mapped changes in protein–protein interactions associated with NF-κB pathway, a regulator of inflammation and immunity that is associated with tumorigenesis of luminal A breast cancer. Identified and further validated interaction network serves as the first but comprehensive data source for further characterization of NF-κB interactions with potential therapeutic implications in tumors with high NF-κB activity.

## Data Availability

The raw mass spectrometry proteomics data for co-fractionation experiments, immunoprecipitation, and total proteome analysis have been deposited to the ProteomeXchange Consortium via the Proteomics Identifications (PRIDE) partner repository (http://www.ebi.ac.uk/pride/archive/) with the dataset identifier PXD040125. Annotated spectra from the co-fractionation experiments are available through MS-Viewer https://msviewer.ucsf.edu/prospector/cgi-bin/msform.cgi?form=msviewer (search key: 5jxjp53vgu for SEC-SILAC dataset; kwq4d0drkz for SEC-LFQ dataset; hf74rtcts0 for SEC-34 fraction dataset). The co-fractionation data have been deposited in the CEDAR database (120) (www3.cmbi.umcn.nl/cedar/, accession number CRX48).

## Supplemental Data

This article contains supplementary data (21, 29, 33, 34, 35, 36, 37, 38, 39, 40, 41, 42, 43, 44, 45, 46, 47, 48, 49, 50, 51, 121, 122, 123, 124, 125, 126, 127, 128, 129, 130, 131, 132, 133, 134, 135, 136, 137, 138, 139, 140, 141, 142, 143, 144, 145, 146, 147, 148, 149, 150, 151, 152, 153, 154, 155, 156, 157, 158, 159).

## Conflicts of Interests

The authors declare that they have no conflicts of interest with the contents of this article.
