# Supplementary material for: Global Interactome Mapping Reveals Pro-tumorigenic Interactions of NF-κB in Breast Cancer
Source: Mol Cell Proteomics. 2024 Feb 28;23(4):100744. doi: 10.1016/j.mcpro.2024.100744 (PMC10988130; doi:10.1016/j.mcpro.2024.100744)
Supplement: Supplemental Information [file mmc11.docx]

**Supplementary Information**

**Global interactome mapping reveals pro-tumorigenic interactions of NF-κB in breast cancer**

Petr Lapcik, R. Greg Stacey, David Potesil, Leonard J. Foster, and Pavel Bouchal^*^

*For correspondence: Pavel Bouchal, [bouchal@chemi.muni.cz](mailto:bouchal@chemi.muni.cz)

**List of supplementary information in this file**

**Supplementary Figures and tables**

**Fig.S1** Detection of presence of the FLAG-tagged NFKBIA fusion protein by SDS-PAGE and Western blotting. (page S3)

**Fig. S2** Comparison of NF-κB inhibited and NF-κB uninhibited protein-protein interaction networks detected by SEC-PCP-SILAC. (page S4)

**Fig. S3** Comparison of NF-κB inhibited and NF-κB uninhibited protein-protein interaction networks detected by SEC-PCP-SILAC focused on RELA protein. (page S5)

**Fig. S4** Co-elution of NF-κB transcription factors and IκB proteins in SEC-label-free fractions. Representative MS/MS spectra and extracted ion current chromatograms of peptides from NFKBIA protein in the SEC-label-free fractions. (page S6)

**Fig. S5** Co-elution of NF-κB factors and proteins co-eluting with RELA across 34 SEC fractions. (page S7)

**Tab. S1** Overview of the proteins conducted to the protein complex prediction using AlphaPulldown pipeline. (page S8)

**Tab. S2** Detailed characterization of the protein complexes predicted by AlphaPulldown. (page S8)

**Fig.S6** Verification of presence of the FLAG-tagged NFKBIA fusion protein in cells used for immunoprecipitation experiment using SDS-PAGE and Western blotting. (page S9)

**Fig.S7** Extracted ion current chromatograms of NFKBIA precursors and comparison of NFKBIA precursor profiles in the total proteome experiment. (page S10)

**Supplementary Methods**

Western blotting (page S11)

Protein complex analysis using size-exclusion chromatography and mass spectrometry in label-free wild type MCF-7 cells (page S11-13)

**List of Supplementary Files**

Supplementary File 1 - The m/z precursor range windows used for LC-MS/MS measurements in DIA mode for immunoprecipitation and total proteome experiments.

Supplementary File 2 - Mass spectrometry protein group level data from SEC-PCP-SILAC experiment consisting of 80 SEC fractions from MCF-7 lysates with inhibited NF-κB (SILAC heavy) or uninhibited NF-κB (SILAC medium) pathway.

Supplementary File 3 - Mass spectrometry protein group level data from SEC-label-free experiment consisting of 40 SEC fractions from MCF-7 lysates with inhibited NF-κB pathway (fraction 1-40) and 40 SEC fractions from MCF-7 lysates with uninhibited NF-κB pathway (fraction 41-80).

Supplementary File 4 - List of protein-protein interactions detected by SEC-PCP-SILAC and SEC-label-free experiments in MCF-7 cells with inhibited NF-κB pathway. Legend: Unique interaction - interaction is present in NF-κB inhibited interaction network only; List of protein-protein interactions detected by SEC-PCP-SILAC and SEC-label-free experiments in MCF-7 cells with uninhibited NF-κB pathway. Legend: Unique interaction - interaction is present in NF-κB uninhibited interaction network only.

Supplementary File 5 - List of significantly enriched GO pathways for proteins participating in unique interactions from SEC-PCP-SILAC experiment; Interactions of non-proteasomal proteins from "NIK/NF-kappaB signaling" pathway detected in SEC-PCP-SILAC experiment.

Supplementary File 6 - Known NF-κB target genes with its protein products present in our SEC-PCP-SILAC and SEC-label-free interaction data; Interactions of proteins produced by NF-κB target genes detected by SEC-PCP-SILAC and SEC-label-free in NF-κB inhibited and NF-κB uninhibited MCF-7 cells. Legend: Unique interaction - interaction is present in inhibited or uninhibited interaction network only.

Supplementary File 7 - List of known RELA interactors from BioGRID, CORUM, HINT, HIPPIE, IID, InBioMap, MENTHA, MINT and PINA databases.

Supplementary File 8 - Mass spectrometry protein group level data from label-free SEC-LC-MS/MS experiment consisting of 34 SEC fractions from MCF-7 lysates.

Supplementary File 9 - Mass spectrometry protein group level data from immunoprecipitation experiment - Spectronaut output. Legend: Inhibited=cells transfected with full pCMV4-FLAG-ΔN IκBα plasmid, Uninhibited=cells transfected with empty pCMV4 plasmid, RELA=proteins bound on anti-RELA antibody, ctrl=proteins bound on control IgG; Mass spectrometry protein group level output from Spectronaut software of protein sequence coverage. Legend: PG.Coverage - coverage of protein sequence in one run, PG.Coverage (Global) - coverage of protein sequence across all runs; Mass spectrometry protein group level data from immunoprecipitation experiment - comparison between conditions. Legend: Inhibited=cells transfected with full pCMV4-FLAG-ΔN IκBα plasmid, Uninhibited=cells transfected with empty pCMV4 plasmid, RELA=proteins bound on anti-RELA antibody, ctrl=proteins bound on control IgG; Lists of RELA-interacting proteins identified in the immunoprecipitation experiment; Results of GSEA analysis of all quantified proteins in RELA immunoprecipitates from NF-κB inhibited or uninhibited cells.

Supplementary File 10 - Mass spectrometry protein group level data for DIA experiment evaluating changes in total proteome after NF-κB inhibition in MCF-7 cells. Legend: inhibited=cells transfected with full pCMV4-FLAG-ΔN IκBα plasmid, control=cells transfected with empty pCMV4 plasmid; Mass spectrometry protein group level output from Spectronaut software of protein sequence coverage. Legend: PG.Coverage - coverage of protein sequence in one run, PG.Coverage (Global) - coverage of protein sequence across all runs; List of significantly (q-value < 0.05) up-regulated (Log2FC > 0.58) proteins after NF-κB inhibition in the total proteome analysis; List of significantly (q-value < 0.05) down-regulated (Log2FC < -0.58) proteins after NF-κB inhibition in the total proteome analysis.

**Supplementary Figures and Tables**


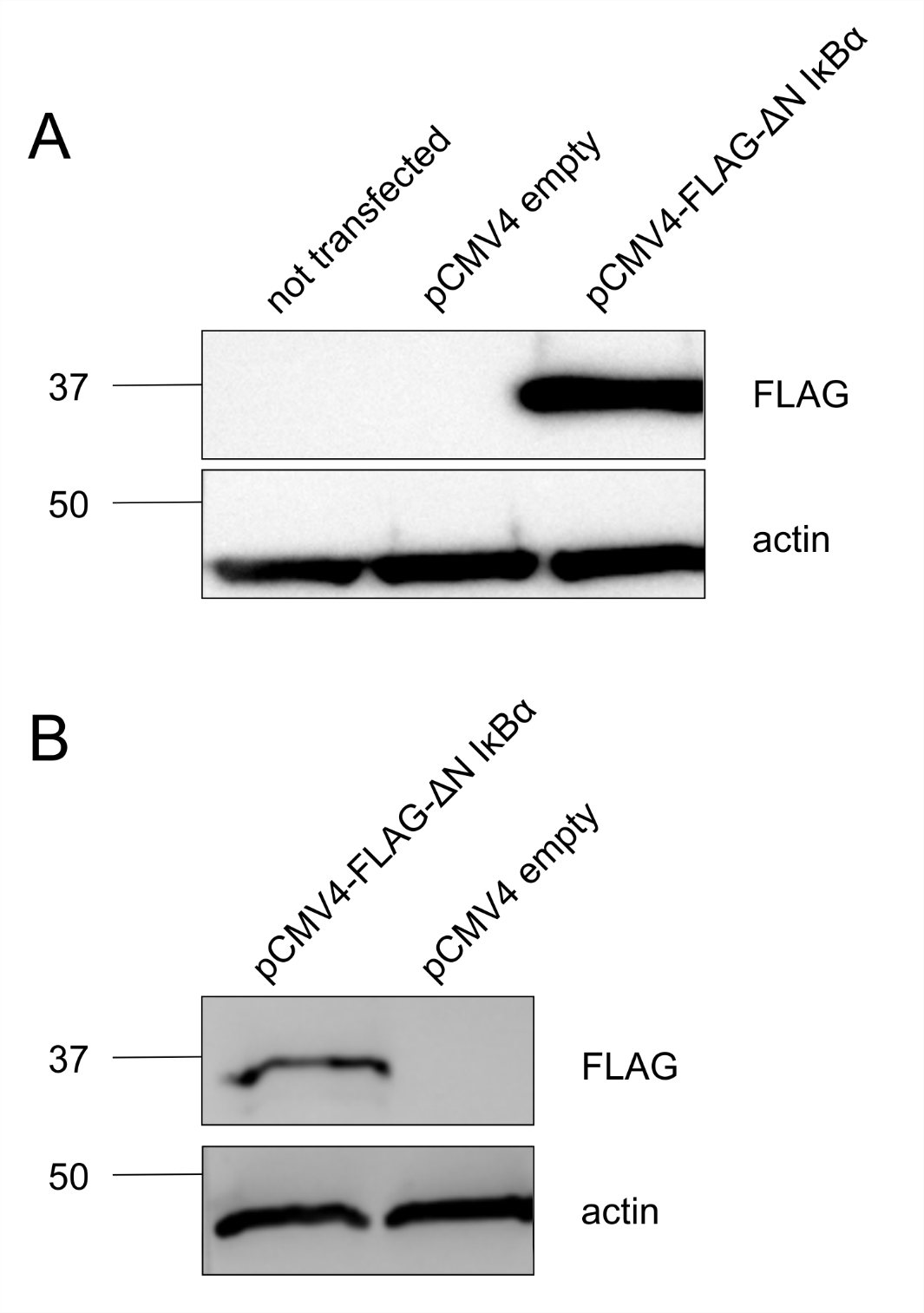


**Fig.S1** Presence of the FLAG-tagged NFKBIA fusion protein in cells transfected with FLAG-NFKBIA encoding plasmid and control cells transfected with empty pCMV4 plasmid of the **A** SEC-PCP-SILAC replicate, and **B** of the SEC-label-free replicate, was verified by SDS-PAGE and Western blotting with mouse anti-FLAG and mouse anti-β-actin antibodies, which served as a loading control.


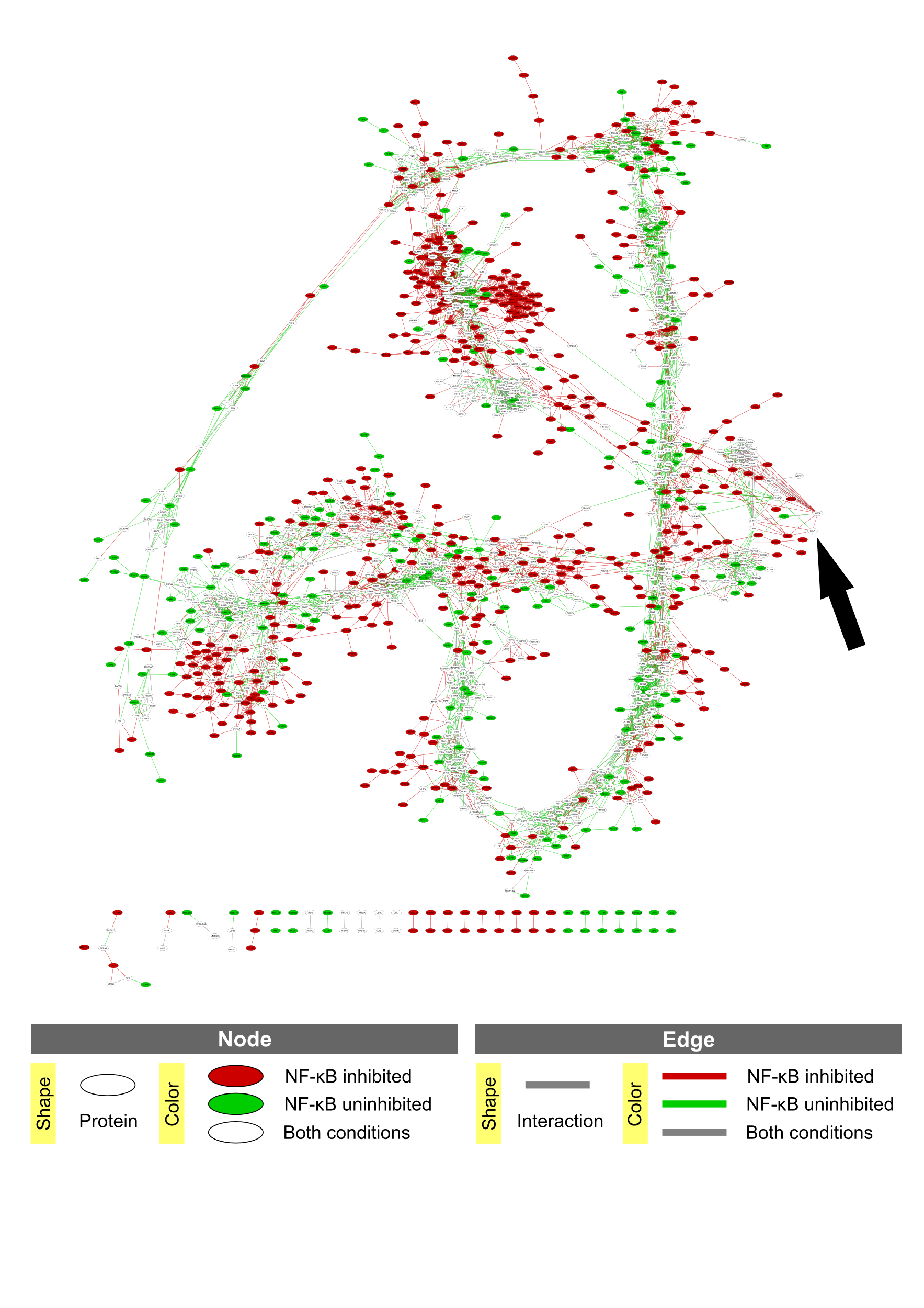


**Fig. S2** Comparison of NF-κB inhibited and NF-κB uninhibited protein-protein interaction networks detected by SEC-PCP-SILAC. RELA is marked with an arrow.


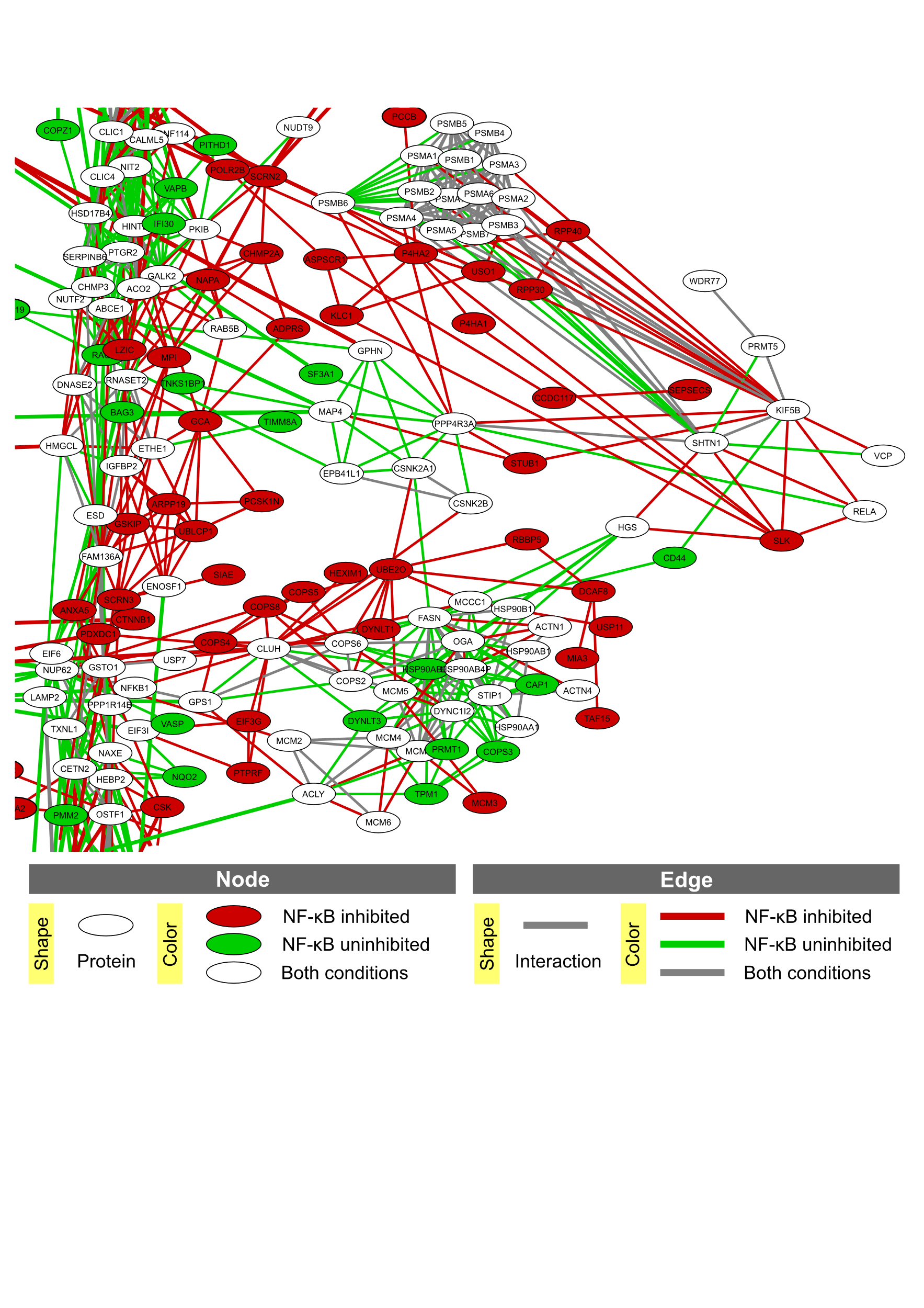


**Fig. S3** Comparison of NF-κB inhibited and NF-κB uninhibited protein-protein interaction networks detected by SEC-PCP-SILAC focused on RELA protein and its interaction partners.


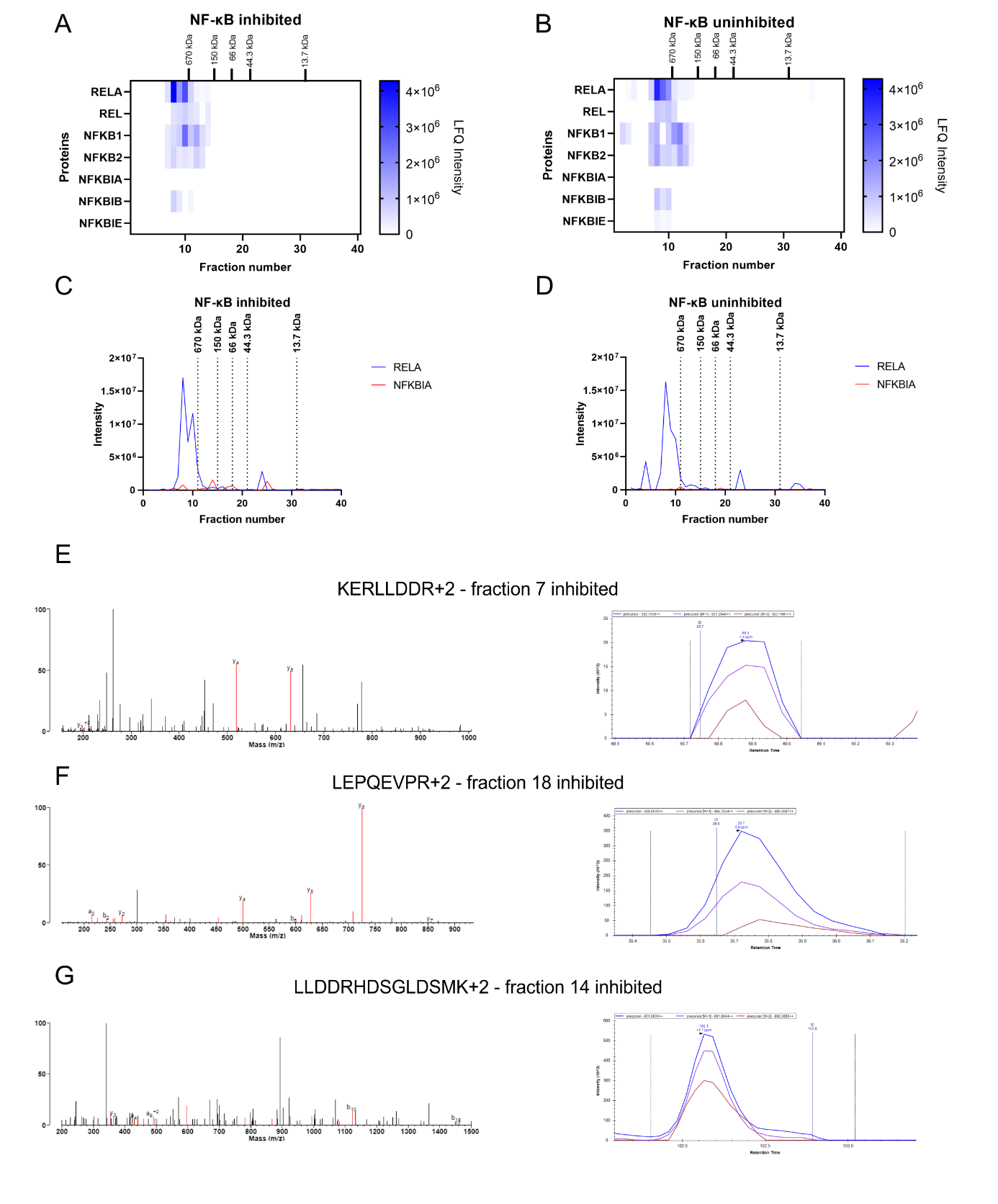


**Fig. S4** Co-elution of NF-κB transcription factors and IκB proteins in SEC-label-free fractions of **A** NF-κB inhibited and **B** NF-κB uninhibited cells. Co-elution of RELA and NFKBIA in SEC-label-free fractions of **C** NF-κB inhibited and **D** NF-κB uninhibited cells. **E-G** Representative MS/MS spectra and extracted ion chromatograms of peptides from NFKBIA protein in the SEC-label-free fractions from cell lysates with inhibited NF-κB.


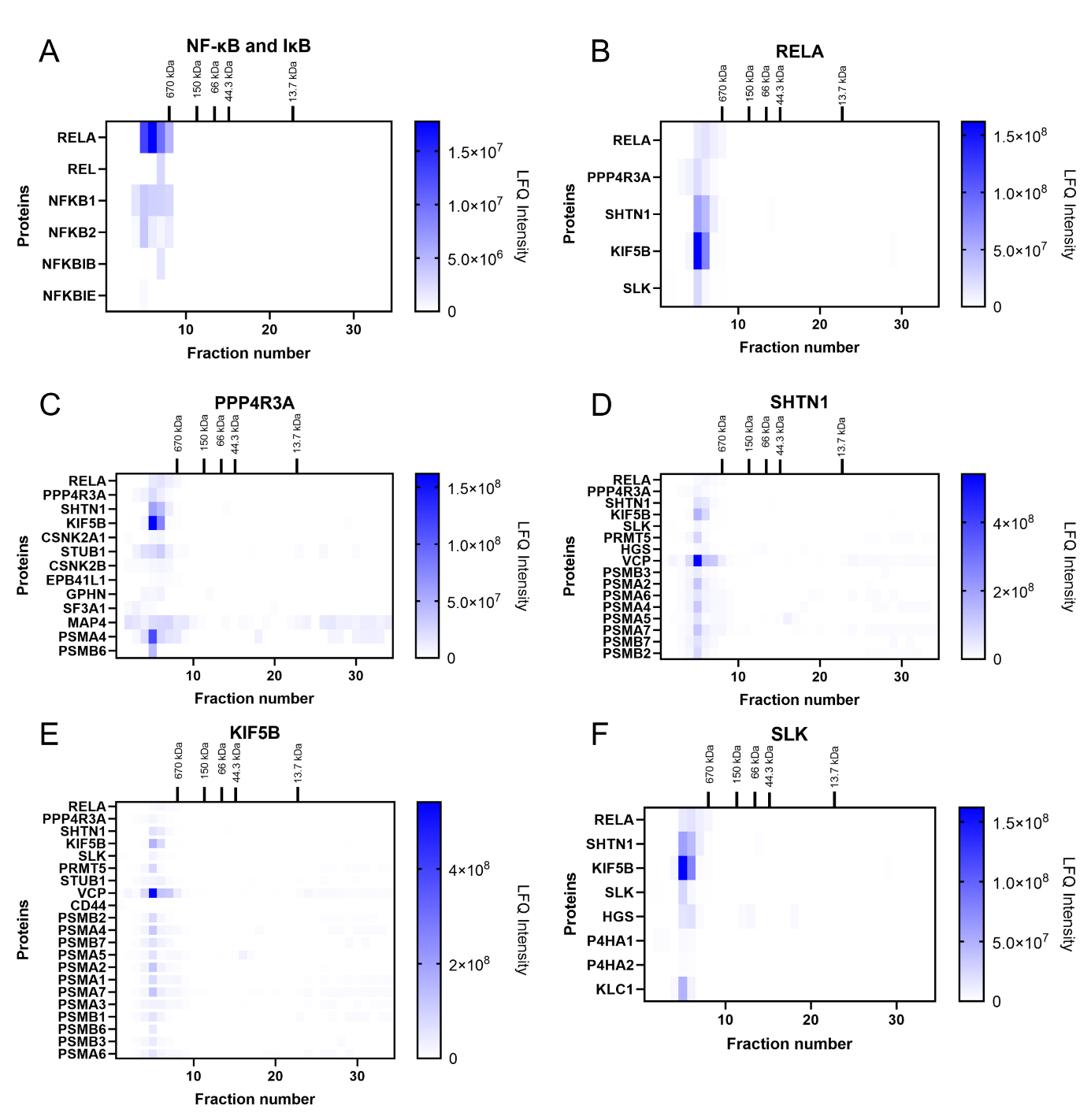


**Fig. S5** Co-elution of **A** NF-κB factors and IκB proteins, and SEC-PCP-SILAC interaction partners of **B** RELA, **C** PPP4R3A, **D** SHTN1, **E** KIF5B, and **F** SLK across 34 SEC fractions of protein complexes from unlabeled wild-type MCF-7 cells.

**Tab. S1** Overview of the proteins conducted to the protein complex prediction using AlphaPulldown pipeline.

| **Gene** | **Protein description** | **Uniprot ID** | **#AAs** | **Exp. Structure** |
| --- | --- | --- | --- | --- |
| CSNK2A1 | **Casein kinase II subunit alpha** | P68400-1 | 391 | X-ray: 1-329 |
| KIF5B | **Kinesin-1 heavy chain** | P33176 | 963 | X-ray, EM: 1-349 |
| PPP4R3A | **Serine/threonine-protein phosphatase 4 regulatory subunit 3A** | Q6IN85-1 | 833 | X-ray: 1-117 |
| PRMT5 | **Protein arginine N-methyltransferase 5** | O14744-1 | 637 | X-ray: 1-637 |
| SHTN1 | **Shootin-1** | A0MZ66-1 | 631 | none |

**Tab. S2** Detailed characterization of the protein complexes predicted by AlphaPulldown.

|  |  | **PAE cutoff** | | **Interface Residues** | | | |  |  |
| --- | --- | --- | --- | --- | --- | --- | --- | --- | --- |
| **Protein A** | **Protein B** | **5 Å** | **10 Å** | **Total** | **Polar [%]** | **Hydrophob [%]** | **Charged [%]** | **HB** | **SB** |
| CSNK2A1 | PPP4R3A |  | x | 44 | 34.6 | 19.2 | 23.1 | 16 | 12 |
| PPP4R3A | SHTN1 |  | x | 73 | 24.3 | 37.9 | 24.8 | 30 | 7 |
| KIF5B | PRMT5 |  | x | 206 | 18.2 | 27.3 | 52.3 | 58 | 15 |
| KIF5B | SHTN1 | x | x | 150 | 41.1 | 24.7 | 28.8 | 35 | 20 |

Total: number of residues at the interface. Polar: percentage of polar residues (Ser, Thr, Asn, Gln, His and Tyr) at the interface. Hydrophob: percentage of hydrophobic residues (Ala, Leu, Ile, Val, Phe, Trp, Cys, Met) at the interface. Charged: percentage of charged residues (Asp, Glu, Lys, Arg) at the interface. HB: number of hydrogen bonds in the interface. SB: number of salt bridges at the interface.

**
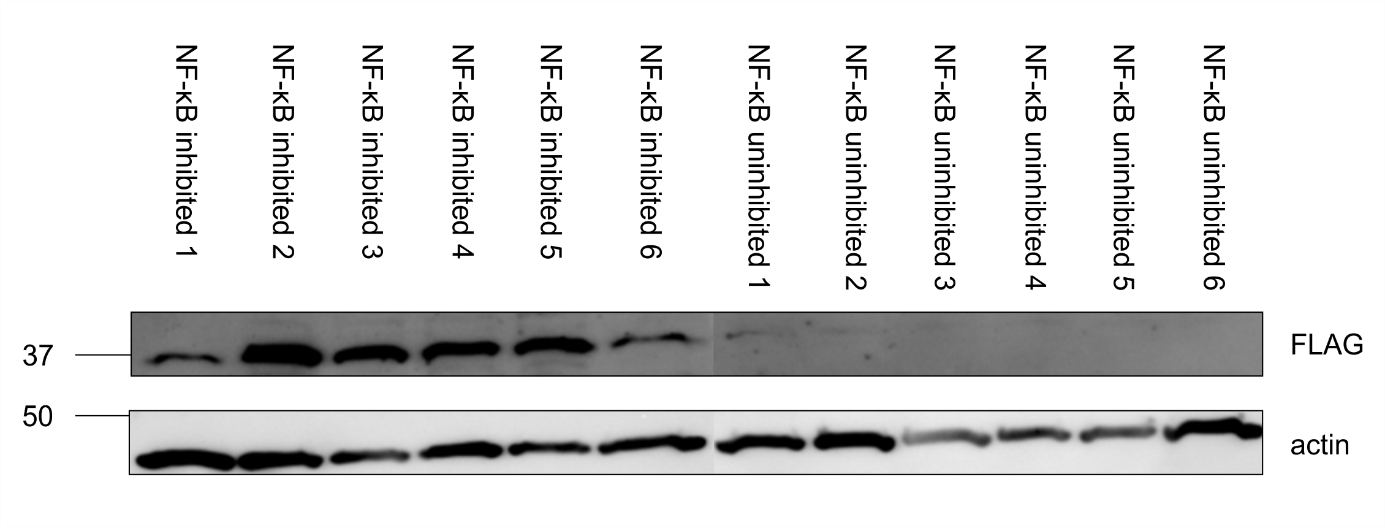
**

**Fig.S6** Presence of the FLAG-tagged NFKBIA fusion protein in cells transfected with FLAG-NFKBIA encoding plasmid and control cells transfected with empty pCMV4 plasmid for the immunoprecipitation experiment, was verified by SDS-PAGE and Western blotting with mouse anti-FLAG and mouse anti-β-actin antibodies, which served as a loading control.


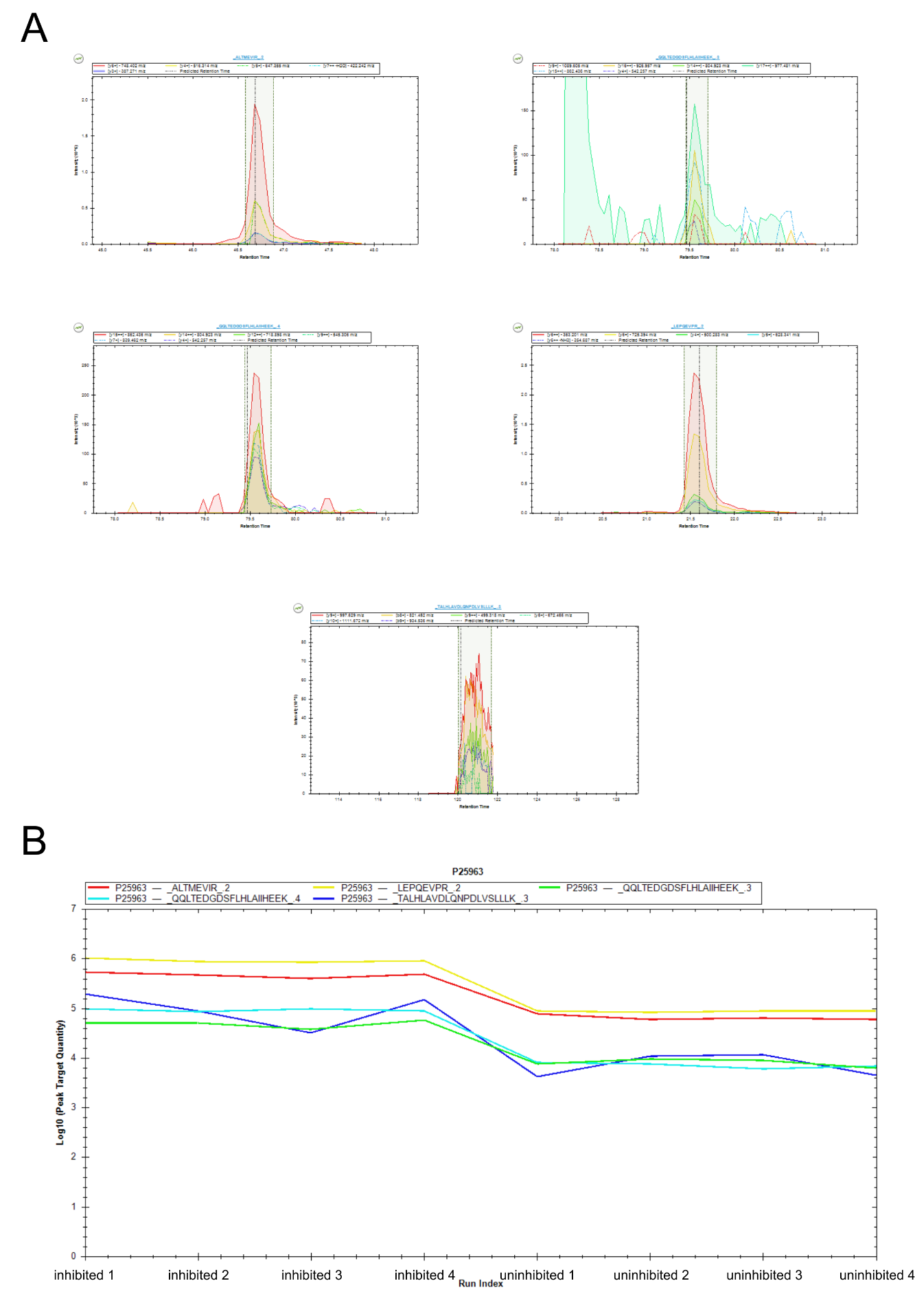


**Fig. S7** **A** Extracted ion current chromatograms of NFKBIA precursors detected in MCF-7 cells with inhibited NF-κB in total proteome experiment. **B** Comparison of NFKBIA precursor profiles in cells with inhibited and uninhibited NF-κB in the total proteome experiment.

**Supplementary Methods**

**Western blotting**

SDS PAGE and western blotting analysis were performed as in (121). For preparation of cell lysates, the cells were twice washed with ice-cold PBS and lysed in 2x Complete Sample Buffer (0.125 M Tris/HCl pH 6.8, 20% glycerol, 0.0005% bromophenol blue, 4% sodium dodecyl sulfate (SDS), 10% mercaptoethanol) for 10 min at 95°C. Lysates were cooled on ice for 3 min. Proteins were separated by SDS–PAGE and transferred onto PVDF membranes with 1x blotting buffer (24.8 mM Tris, 193 mM glycine, 20% methanol). Proteins were detected using mouse anti-FLAG antibody (1:1000, Sigma-Aldrich) and mouse anti-β-Actin antibody (1:1000, Sigma-Aldrich) as a loading control and mouse-specific secondary HRP-coupled antibody (RAMPx, 1:1000, Dako).

**Protein complex analysis using size-exclusion chromatography and mass spectrometry in label-free wild type MCF-7 cells**

**Cell harvesting and preparation of protein complex samples for SEC analysis**

Breast cancer cell line MCF-7 was grown in complete DMEM media (Dulbecco's Modified Eagle's Medium, Sigma-Aldrich, USA) containing 10% FBS (Fetal Bovine Serum, Sigma-Aldrich, USA) under conditions including 5% CO_2_ and 37°C. Cells were passaged after reaching 80% confluence. Growth medium was removed from plates, cells were washed with 0.5% EDTA in phosphate buffered saline (PBS; 0.137 M NaCl; 2.68 mM KCl; 1.47 mM KH_2_PO_4_; 6.45 mM Na_2_HPO_4_) and cells were treated with 0.125% trypsin solution at 37°C for 3-6 min. Cells were washed with complete medium and transferred on new plates. For the SEC-MS experiment, eight 15 cm dishes with MCF-7 cells were grown to about 90% confluency. Medium was removed and cells were washed three times with ice cold 1x PBS. Dishes with cells were then moved to ice. Chilled cOmplete™, EDTA-free Protease Inhibitor Cocktail (Roche) in the SEC mobile phase (50 mM Tris, 50 mM KCl and 50 mM Na-acetate, pH 7.2) was added to the plates. Cells were then scraped from plates and mixed. Subsequently, cells were transferred to a Dounce tissue grinder and were lysed for 4 minutes. Cell lysis was verified under a microscope by trypan blue staining. The homogenates were then centrifuged at 100,000g and 4°C for 15 min and supernatants were concentrated using the 100 kDa molecular weight cutoff spin columns (Sartorius Vivaspin). Sample was further subjected to SEC fractionation.

**Fractionation of protein complexes using SEC**

Separation of protein complexes was performed on Agilent 1100 liquid chromatograph (Agilent Technologies, USA) using 50 x ProSEC 300S 50 x 7.5 mm pre-column (Agilent Technologies, USA) and two serially connected ProSEC 300S 300 x 7.5 mm columns (Agilent Technologies, USA). System was first washed with SEC mobile phase and then 190 μl of sample was loaded. Separation was carried out at a flow rate of 0.5 ml/min at 4°C. Fractions were collected from 20 to 30 minute with 10 fractions (1 fraction/min) and from 30 to 51 minute with 24 fractions (0.875 fractions/min). Mixture of standards (Protein Standard Mix 15 - 600 kDa, Sigma-Aldrich) with added bovine serum albumin (BSA, 66 kDa, Bio-Rad) was used for calibration of SEC columns. Protein concentrations in all fractions were determined using RC-DC protein assay kit (Bio-Rad). Samples were submitted to trypsin digestion using Filter-Aided Sample Preparation (FASP) method (24) and desalted as previously described (25). Briefly, proteins from the whole combined fractions were transferred to the Microcon filter device, cut-off 30 kDa (Millipore), reduced by tris(2-carboxyethyl)phosphine) (Sigma-Aldrich) in 8 M urea in 0.1 M Tris/HCl, pH 8.5, alkylated using iodoacetamide (Sigma-Aldrich), digested by trypsin (Promega) with the ratio trypsin:protein 1:30 at 37°C overnight, and resulting peptides were desalted on MicroSpin columns C18 (Nest Group).

**LC-MS/MS identification of peptides in DDA mode**

LC-MS/MS analysis of peptide samples was performed using the RSLCnano system (Thermo Fisher Scientific, Waltham, MA, USA) online connected to Impact II Ultra-High Resolution Qq-Time-Of-Flight (Bruker, Bremen, Germany) mass spectrometer. Peptides were preconcentrated online on a 100 μm × 30 mm trapping column packed with 3.5-μm X-Bridge BEH 130 C18 (Waters, Milford, MA, USA) prior to LC separation. Equilibration of the trapping and analytical column was performed before the sample injection. Peptides were separated using an Acclaim Pepmap100 C18 column (3 μm particles, 75 μm × 500 mm, Thermo Fisher Scientific) using the following LC gradient (mobile phase A: 0.1% FA (formic acid) in water, mobile phase B: 0.1% FA in 80% acetonitrile: 300 nl/min; 40°C): Elution gradient started at 1% of mobile phase B, which increased to 56% over 120 minutes nonlinearly (40min: 14%, 80min: 30%, 120min: 56%) followed by the system wash phase. Analytical column outlet was connected to the CaptiveSpray nanoBooster ion source (Bruker, Bremen, Germany). NanoBooster was filled with acetonitrile. MS and MS/MS spectra were measured in data-dependent mode with 3 s long cycle. The mass range was set to 150-2200 m/z with precursors selection from 300 to 2000 m/z. Measurement frequency of MS and MS/MS scans were 2 Hz and 4-16 Hz (depending on the precursor intensity).

**Processing of LC-MS/MS data**

Protein identification and quantification was performed in MaxQuant 1.6.2.10. Database search was performed against human UniProt/SwissProt database (version 2019_07 downloaded on 2019-09-16, 20431 sequences) using standard settings for Bruker qTOF mass spectrometer. Enzyme specificity was set to trypsin/P, two missed cleavages were allowed, fixed modifications were set to carbamidomethylation (C), and variable modifications were set to oxidation (M) and acetylation (protein N-terminus). Match between runs was not activated. Label-free quantification was activated. MS/MS mass tolerance was 40 ppm (TOF). FDR at peptide and protein levels were set to 0.01. Potential contaminants, reverse hits and peptides identified only based on posttranslational modifications were removed from the search result. Co-elution graphs were visualized in GraphPad Prism (version 9.0.1) software.
